# Immune–tumor cell ligand–receptor axes driving metabolic reprogramming and therapeutic resistance in cancer

**DOI:** 10.3389/fimmu.2026.1864068

**Published:** 2026-06-16

**Authors:** Hailin Zhu, Wang Yi, Yujie Wu, Rong Li, Boxuan Zhou

**Affiliations:** 1 Department of Pathology, The Affiliated Cancer Hospital Of Gannan Medical University, Ganzhou, China; 2 Department of Breast Disease Center, The First Affiliated Hospital of Nanchang University, The First Clinical Medical College of Nanchang University, Jiangxi Medical College, Nanchang, China; 3 Hepatobiliary and Pancreatic Surgery, The Second Affiliated Hospital of Nanchang University, Second Clinical Medical College of Nanchang University, Jiangxi Medical College, Nanchang, China

**Keywords:** drug resistance, immune checkpoint, immune–tumor communication, lactate signaling, ligand–receptor axis, metabolic reprogramming, spatial omics, tumor-associated macrophages

## Abstract

Therapeutic resistance remains a major barrier to durable cancer control and cannot be fully explained by tumor-intrinsic genetic and epigenetic alterations alone. Increasing evidence indicates that resistance emerges within a dynamic tumor microenvironment in which immune cells actively instruct tumor cell behavior through ligand–receptor (LR) signaling. These immune–tumor communication axes link inflammatory cues, checkpoint-associated signals, chemokine networks, and metabolite-derived messages to adaptive tumor phenotypes. In particular, these axes may contribute to metabolic reprogramming across glucose, lipid, amino acid, and redox pathways, thereby supporting tumor-cell proliferation, therapeutic stress tolerance, and immune evasion. Lactate-centered signaling, macrophage-derived cytokine and chemokine axes, and checkpoint-associated pathways such as PD-L1-related signaling have emerged as major regulators of this process. These LR-mediated circuits are increasingly associated with tumor metabolic remodeling, immune suppression, phenotypic plasticity, and reduced responsiveness to chemotherapy and immune checkpoint blockade. Recent advances in single-cell transcriptomics, spatial omics, multiplex imaging, metabolomics, and computational modeling are accelerating the mapping of these communication networks *in situ* and revealing clinically relevant resistance niches. In this review, we synthesize current evidence on how immune–tumor cell LR axes drive metabolic adaptation and therapeutic resistance across cancers, discuss the technologies enabling their dissection, and highlight their translational potential as biomarkers and therapeutic targets. Understanding these communication systems may provide new opportunities to disrupt resistant tumor ecosystems and improve the durability of cancer therapy.

## Highlights

- Immune–tumor ligand–receptor axes actively drive tumor metabolic reprogramming rather than merely accompanying resistance.
- Lactate signaling, macrophage-derived inflammatory cues, and checkpoint-associated pathways integrate immune suppression with metabolic adaptation and therapy failure.
- Targeting immune–tumor communication together with metabolic dependencies may offer a rational strategy to overcome resistant tumor niches.

## 1 Introduction

Therapeutic resistance remains one of the most formidable barriers to durable cancer control (1–4). Despite substantial advances in chemotherapy, targeted therapy, radiotherapy, and immunotherapy, many tumors either fail to respond initially or eventually acquire resistance during treatment. Traditionally, resistance has been interpreted largely through a tumor-intrinsic lens, with emphasis placed on genetic mutations, clonal selection, epigenetic alterations, lineage plasticity, and adaptive activation of compensatory signaling pathways (5–8). These mechanisms are undoubtedly important, yet they are insufficient to explain the full complexity, reversibility, and context dependency of resistance observed in clinical and experimental settings.

Cancer cells do not evolve in isolation. Rather, they exist within a highly dynamic tumor microenvironment in which immune cells, stromal components, vascular elements, extracellular matrix (ECM), and soluble mediators collectively shape tumor behavior (9, 10). Continuous exposure to inflammatory cytokines, chemokines, growth factors, metabolic by-products, and matrix-derived signals imposes strong selective and adaptive pressures on tumor cells. Under therapeutic stress, these microenvironmental cues can become even more influential, promoting survival programs that allow malignant cells to evade cell death, maintain fitness, and enter drug-tolerant states. Thus, therapeutic resistance should be viewed not only as a consequence of tumor cell-autonomous evolution, but also as an emergent property of reciprocal communication between tumor cells and their surrounding cellular ecosystem. Among the multiple layers of tumor–microenvironment crosstalk, ligand–receptor (LR) interactions represent one of the most direct and functionally consequential modes of communication (11, 12). Through receptor-mediated sensing of extracellular signals, tumor cells can decode cues derived from immune cells, including cytokines, chemokines, checkpoint ligands, and inflammatory metabolites, and translate them into intracellular programs that promote adaptation. These programs often converge on metabolic remodeling, enabling tumor cells to rewire glucose, lipid, amino acid, and redox metabolism in ways that support proliferation, stress tolerance, immune evasion, and resistance to therapy (13–15). In this context, metabolic reprogramming is not merely a passive by-product of malignant transformation, but a highly regulated adaptive process shaped by extrinsic signals from the immune microenvironment.

Importantly, therapy itself can intensify this process. Cytotoxic damage, targeted pathway inhibition, and immune pressure all reshape the tumor ecosystem, alter immune cell composition and activation states, and change the availability of extracellular ligands and metabolites. As a result, treatment may inadvertently reinforce signaling circuits that help residual cancer cells survive. Understanding how extrinsic microenvironmental signals, particularly those derived from immune cells, contribute to metabolic adaptation is therefore essential for explaining why many tumors persist despite apparently effective initial therapy (16–18).

A growing body of evidence suggests that immune cells should not be viewed solely as cytotoxic effectors or suppressive bystanders, but also as active senders of instructive signals that shape tumor cell phenotypes (19–21). Macrophages, neutrophils, myeloid-derived suppressor cells, dendritic cells, regulatory T cells, and dysfunctional effector lymphocytes release a broad array of ligands, including cytokines, chemokines, growth factors, checkpoint molecules, lipid mediators, and metabolic cues. These signals are detected by corresponding receptors on tumor cells and can profoundly influence malignant behavior. In parallel, tumor cells themselves are not simply passive targets of immune attack; they are highly plastic signal integrators capable of interpreting, amplifying, and reprogramming their responses to immune-derived inputs. Focusing on immune–tumor cell LR axes provides a useful conceptual framework for understanding this bidirectional relationship. First, LR interactions offer specificity. They identify not only which molecules are present in the microenvironment, but also which cell populations are likely to communicate functionally. Second, LR signaling provides mechanistic directionality, linking extracellular immune cues to intracellular tumor signaling networks such as PI3K/AKT, JAK/STAT3, NF-κB, HIF-1α, mTOR, YAP/TAZ, and SREBP-dependent programs (22, 23). Third, these axes help explain why resistance is often dynamic, reversible, and spatially heterogeneous. Because ligand availability depends on immune cell composition, activation status, localization, and treatment context, LR-dependent tumor phenotypes can change over time rather than remaining fixed. This framework is particularly relevant to cancer metabolism. Many immune-derived signals have been reported to directly or indirectly influence metabolic pathways in tumor cells, including glycolysis, oxidative phosphorylation, lactate handling, fatty acid synthesis and oxidation, amino acid utilization, and antioxidant defense (24–27). Conversely, tumor metabolic states can alter ligand production, receptor expression, and immune cell behavior, generating self-reinforcing feedback loops. For example, inflammatory signaling may enhance glycolysis and lactate secretion, while lactate-rich conditions can in turn promote immune suppression and checkpoint signaling (28). In this way, immune–tumor LR axes serve as coupling systems that connect inflammation, metabolic stress, and therapeutic selection pressure into integrated adaptive circuits. Another reason to center LR signaling is that it offers a more informative framework than studying isolated intracellular pathways alone. Many pathways implicated in resistance, such as AKT, STAT3, or HIF-1α, are activated downstream of multiple upstream cues. Examining them in isolation may describe the endpoint of adaptation, but not the microenvironmental logic that drives it. By contrast, analyzing immune–tumor LR axes allows resistance to be understood at the interface between extracellular signals and intracellular rewiring. This is especially valuable for identifying clinically actionable vulnerabilities, because ligands, receptors, and their downstream metabolic dependencies may all be targetable. Thus, immune–tumor LR axes may represent more than descriptive cell–cell interactions. In selected contexts, they may function as dynamic regulatory modules through which the immune microenvironment influences tumor metabolic plasticity, phenotypic adaptation, and therapeutic outcome. Understanding these axes may therefore provide a unifying framework for linking tumor immunology, cancer metabolism, and treatment resistance.

In this review, we focus on immune–tumor ligand–receptor (LR) communication as a directional mechanism linking immune signaling, metabolic reprogramming, and therapeutic resistance. Rather than treating cancer immunometabolism, immune escape, and TME-driven resistance as separate processes, we organize them into an integrated sender–receiver framework in which immune-derived ligands, tumor-cell receptors, and metabolite-associated receptor signaling remodel glucose metabolism, lipid programs, amino acid dependency, and redox adaptation under therapeutic pressure. This perspective distinguishes the review from prior discussions by emphasizing three points: first, LR axes define which immune populations instruct specific tumor metabolic states; second, established mediators such as lactate, TAM-derived IL-6/CCL2, PD-L1, STAT3, AKT, and HIF-1α can be understood as interconnected communication circuits rather than isolated mechanisms; and third, LR-driven resistant niches are spatially organized, dynamically reshaped by therapy, and potentially reversible. We further discuss how single-cell transcriptomics, spatial omics, multiplex imaging, metabolomics, and computational LR inference can identify context-specific communication axes, while emphasizing their translational potential as biomarkers and targets for rational combination therapy. Overall, this review provides a conceptual and mechanistic synthesis of how immune–tumor LR axes convert extracellular immune cues into tumor-intrinsic metabolic programs that sustain immune evasion, drug tolerance, and therapy resistance in a tumor-type- and treatment-context-dependent manner.

## 2 Conceptual framework of immune–tumor cell ligand–receptor axes

### 2.1 Defining immune–tumor ligand–receptor interactions

Intercellular communication within the tumor microenvironment is mediated through multiple mechanisms, including soluble factors, extracellular vesicles, direct cell–cell contact, and matrix-associated signaling (29, 30). Among these, ligand–receptor interactions represent one of the most fundamental and tractable modes through which immune cells and tumor cells exchange information. In the context of cancer, immune–tumor ligand–receptor (LR) interactions can be broadly defined as signaling events in which ligands produced, released, displayed, or induced by immune cells engage cognate receptors on tumor cells, or, conversely, tumor-derived ligands and metabolites act on receptors expressed by immune cells to shape reciprocal responses. Although these directions are biologically intertwined, the present review places primary emphasis on how such LR-mediated communication reprograms tumor cell metabolism and contributes to therapeutic resistance.

A key advantage of the LR framework is that it shifts the analysis of tumor progression from static molecular inventories to dynamic signaling relationships. Rather than simply cataloging cytokines, chemokines, immune checkpoints, or metabolites that are enriched in the tumor milieu, LR analysis asks which signaling pairs are functionally connected, which cell types are acting as senders and receivers, and what phenotypic outputs emerge from these exchanges. This is especially important in tumors, where the abundance of a given ligand or receptor alone may not predict biological effect unless the spatial proximity, receptor availability, downstream competency, and treatment context are also considered. Thus, immune–tumor LR interactions should be understood not merely as molecular co-expression patterns, but as context-dependent communication modules that encode directional information across cell populations.

In practical terms, immune–tumor LR interactions can be grouped into several conceptual classes. One major category involves immune-derived soluble ligands acting on tumor cell receptors. These include inflammatory cytokines such as IL-6, TNF-α, and TGF-β; chemokines such as CCL2, CXCL family members, and related receptor-mediated cues; and growth or stress signals generated by myeloid and lymphoid populations (31). A second category includes surface-bound or contact-dependent interactions, such as checkpoint ligands, co-stimulatory molecules, Notch ligands, and adhesion-related receptors, which often operate at the immune synapse or in juxtacrine niches within the tumor tissue (32–34). A third category, increasingly relevant to cancer metabolism, involves metabolite-associated receptor signaling, in which metabolic products such as lactate act as signaling entities rather than inert waste, engaging receptors that alter tumor–immune dynamics and reinforce adaptive phenotypes. These classes are not mutually exclusive; instead, they often cooperate to form layered communication networks.

Importantly, immune–tumor LR interactions are not uniformly suppressive or stimulatory. Their biological meaning depends on the identity and state of both signaling partners. A ligand released by activated cytotoxic immune cells may promote tumor elimination in one setting, while the same or a related signaling module, when chronically present in a myeloid-rich or hypoxic niche, may instead drive tumor adaptation and immune escape (35–38). Similarly, tumor cell receptors are not passive conduits. Receptor abundance, post-translational modification, membrane localization, internalization dynamics, and coupling to intracellular signaling machinery all determine how an extracellular signal is interpreted. In resistant tumors, receptors may become amplified, ectopically expressed, or preferentially wired to survival and metabolic pathways, thereby converting microenvironmental signals into drug-tolerant phenotypes. Another important aspect of this framework is bidirectionality. Although the title of this review emphasizes immune–tumor axes, tumor cells are not simply recipients of immune instruction. Tumor-derived ligands, metabolites, and checkpoint molecules can profoundly reshape immune cell function, altering recruitment, polarization, activation, and exhaustion states. This reciprocal signaling creates feedback loops in which tumor metabolism and immune behavior co-evolve. For example, a tumor cell that adopts glycolytic metabolism may generate a lactate-rich environment that dampens antitumor immunity, while immune suppression further stabilizes the metabolic state that enabled the tumor to persist. In this sense, LR interactions are best viewed not as isolated linear events but as components of adaptive communication circuits.

Therefore, defining immune–tumor LR interactions requires an integrative perspective that considers ligand source, receptor distribution, signal directionality, spatial organization, downstream signaling competence, and phenotypic consequence. Such a framework provides a biologically coherent foundation for understanding how the immune microenvironment drives tumor metabolic plasticity and resistance across diverse therapeutic contexts.

### 2.2 Why ligand–receptor axes are ideal drivers of metabolic plasticity

Metabolic plasticity is a defining feature of aggressive and treatment-refractory tumors. Rather than committing to a single metabolic program, cancer cells continuously reconfigure their use of glucose, lipids, amino acids, mitochondrial respiration, and redox pathways in response to environmental fluctuations and therapeutic stress. To understand how such plasticity arises, it is necessary to identify mechanisms that are rapid, context-sensitive, reversible, and capable of coordinating multiple intracellular pathways simultaneously. Immune–tumor LR signaling fulfills these requirements remarkably well.

First, LR interactions provide a highly efficient means of coupling extracellular environmental change to intracellular metabolic adaptation. Immune cells respond quickly to tissue damage, therapy-induced stress, necrosis, hypoxia, and tumor-derived signals by altering their secretory profiles and activation states (39–41). As these changes occur, the composition of ligands available within the tumor microenvironment shifts accordingly. Tumor cells can sense these altered immune cues through receptors that rapidly activate downstream signaling cascades without requiring new mutational events. This allows malignant cells to adapt metabolically on timescales much shorter than those required for clonal selection, making LR-driven signaling particularly important during early drug tolerance and acute stress responses. Second, LR axes are intrinsically suited to mediate metabolic plasticity because they converge on central signaling nodes that regulate nutrient uptake, biosynthesis, energy production, and survival. Pathways such as PI3K/AKT/mTOR, JAK/STAT3, NF-κB, MAPK, HIF-1α, YAP/TAZ, AMPK, and SREBP are all well positioned to translate receptor engagement into metabolic consequences (42–46). Activation of these pathways can increase glucose transporter expression, stimulate glycolytic enzymes, promote lactate export, alter mitochondrial function, support lipid synthesis, influence fatty acid oxidation, affect amino acid utilization, or reinforce antioxidant defenses. Because many immune-derived ligands feed into these shared signaling hubs, distinct immune cues can generate convergent metabolic outcomes even across different tumor types. Third, LR signaling offers a mechanism for graded and adaptive control, which is essential for plasticity. Tumor metabolism is not simply “on” or “off”; it is dynamically tuned according to nutrient availability, oxygen tension, immune pressure, and therapy exposure. Ligand concentration, receptor density, binding affinity, signal duration, and receptor crosstalk all influence the intensity and persistence of downstream responses. Chronic low-level exposure to inflammatory ligands may promote a stable survival phenotype, whereas transient bursts of signaling may prime tumor cells for later resistance. Likewise, simultaneous activation of multiple LR axes can create combinatorial signaling states that are not predictable from any single pathway alone. This capacity for graded modulation may allow LR-based signaling to participate in the generation of heterogeneous and reversible metabolic states within tumors. Fourth, LR-mediated metabolic plasticity is spatially organized. Not all tumor cells encounter the same signals. Cells located near macrophage-rich niches, hypoxic regions, necrotic cores, invasive fronts, or vascular interfaces are exposed to distinct constellations of ligands and metabolites (47–49). These localized microenvironments may induce or maintain regional metabolic states associated with invasion, dormancy, survival under therapy, or escape from immune surveillance. LR signaling therefore helps explain why metabolic heterogeneity is often spatially patterned and why specific niches can serve as reservoirs of resistant tumor cells. The emergence of spatial transcriptomics and multiplex imaging has made this principle increasingly evident, revealing that receptor-bearing tumor subpopulations often co-localize with ligand-producing immune cells in defined tissue compartments.

Another reason LR axes are powerful drivers of metabolic plasticity is that they readily participate in feedback loops. Metabolic rewiring associated with immune-derived signals may alter the expression of additional ligands, receptors, transporters, and checkpoint molecules, which may in turn further reshape the microenvironment. Glycolytic activation can increase lactate production, acidify the extracellular space, and impair effector immune function. Lipid remodeling may influence membrane receptor composition and signal transduction efficiency. Amino acid depletion or redox adaptation can affect cytokine responsiveness and antigen presentation (50, 51). Through these feedback mechanisms, an initially transient immune-derived signal may become stabilized as a self-reinforcing metabolic state. Importantly, LR-driven metabolic plasticity also provides a conceptual explanation for why resistance can be reversible. In many tumors, drug-tolerant states do not necessarily reflect permanent genetic fixation, but rather adaptive rewiring maintained by continuous exposure to supportive microenvironmental cues. When these cues are removed or therapeutically disrupted, tumor cells may partially re-sensitize. This suggests that extrinsic signaling axes are not merely auxiliary contributors to resistance but active maintenance systems for plastic metabolic states. As a result, targeting ligand–receptor communication may offer a means of collapsing resistance-supportive metabolic configurations before they become genetically entrenched. Taken together, immune–tumor LR axes are well positioned to contribute to metabolic plasticity because they are rapid, reversible, combinatorial, spatially organized, and closely linked to master regulators of cellular metabolism. They provide a mechanistic bridge between microenvironmental change and tumor cell adaptation, thereby helping to explain how malignant cells survive therapeutic assault without relying exclusively on cell-autonomous genomic evolution.

### 2.3 Major phenotypic consequences downstream of ligand–receptor signaling

The biological significance of immune–tumor LR interactions lies not only in the existence of communication itself, but in the downstream phenotypes they generate. In cancer, LR-mediated signaling rarely produces a single isolated effect. Instead, it reshapes multiple interconnected traits that collectively support tumor persistence. Among the most important phenotypic outputs are metabolic rewiring, immune evasion, phenotypic plasticity, and therapeutic resistance. These outcomes are analytically separable, but biologically overlapping, and together they form the core rationale for centering LR signaling in the study of treatment failure.

**Figure 1:**
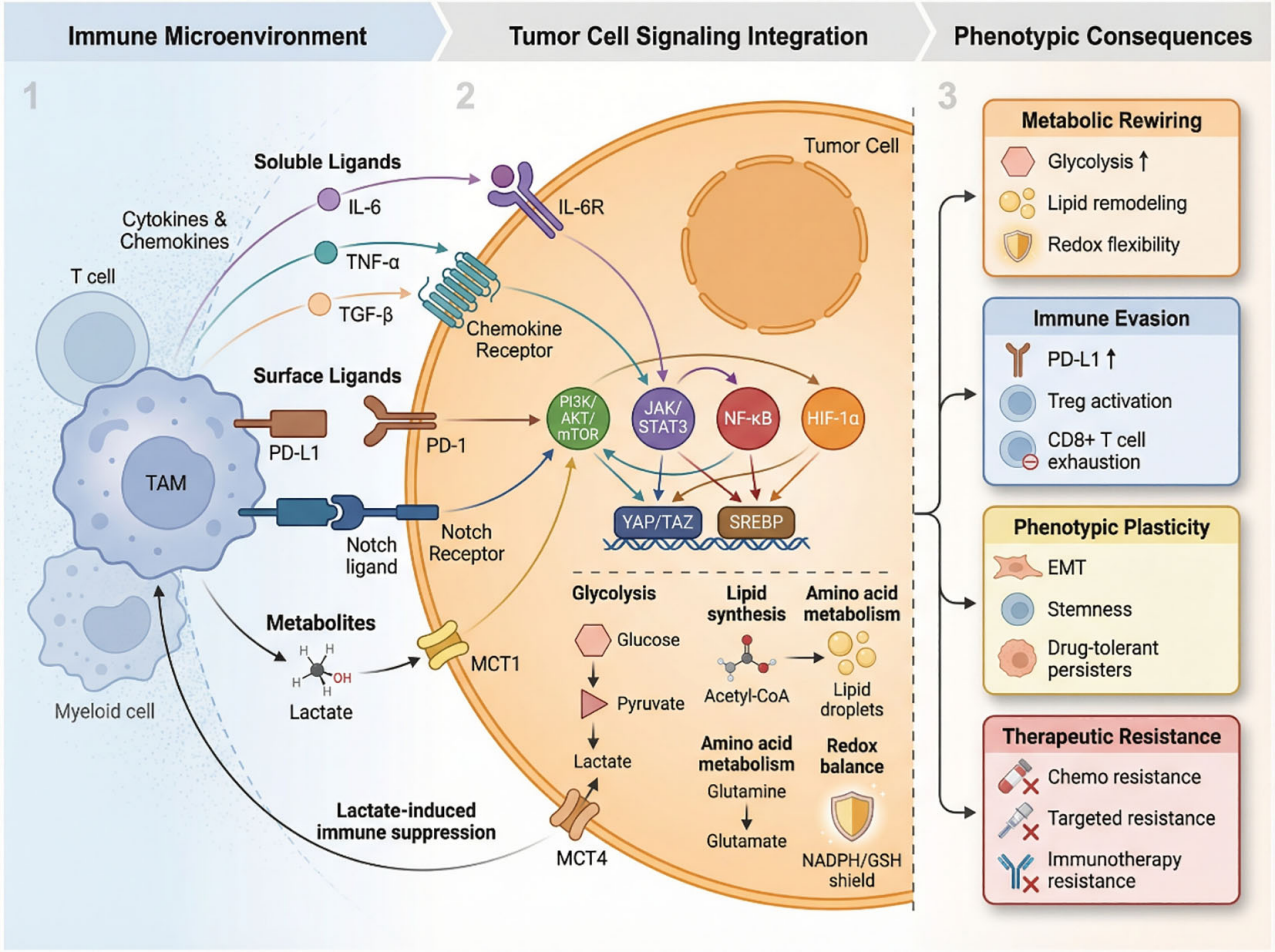
Immune–tumor ligand–receptor axes integrate cytokine, chemokine, checkpoint, and metabolite signals from the tumor microenvironment and transmit them into tumor-cell signaling hubs. These inputs drive metabolic rewiring, immune evasion, phenotypic plasticity, and therapeutic resistance, while lactate-centered feedback further stabilizes an immunosuppressive and resistance-supportive niche.

One major consequence of LR signaling is metabolic rewiring. Immune-derived ligands can promote shifts in glycolysis, oxidative phosphorylation, lipid metabolism, amino acid dependency, and redox homeostasis, enabling tumor cells to cope with nutrient limitation, hypoxia, and cytotoxic stress (52–55). These changes do not simply sustain proliferation; they also allow tumor cells to survive in hostile microenvironments and maintain biosynthetic flexibility under treatment pressure. In many cases, LR-induced metabolic states are not constitutively fixed, but rather context-responsive, allowing tumor cells to switch between energy programs as conditions change. This makes receptor-mediated signaling a particularly effective upstream mechanism for coordinating adaptive metabolic behavior. A second major consequence is immune evasion (56). Metabolic changes driven by LR signaling often feed directly into immune suppression. Glycolytic activation can increase lactate accumulation and acidification, both of which can impair cytotoxic lymphocyte and natural killer cell function. Checkpoint ligand expression may be enhanced downstream of metabolic or inflammatory signaling, thereby reducing antitumor immune activity. Altered lipid metabolism can promote membrane remodeling, receptor stabilization, or secretion of immunoregulatory mediators. Meanwhile, tumor-derived metabolites generated as a result of these changes can act back on immune cells to foster exhaustion, suppress antigen presentation, or recruit immunosuppressive populations such as regulatory T cells and myeloid-derived suppressor cells. Thus, LR signaling often establishes a reciprocal system in which tumor adaptation and immune dysfunction reinforce one another. A third output is phenotypic plasticity, including transitions toward stem-like, mesenchymal-like, invasive, or drug-tolerant states. Many receptor-activated signaling pathways intersect with transcriptional programs that regulate epithelial–mesenchymal transition, dedifferentiation, quiescence, and stress tolerance (57–59). These changes are especially relevant in the setting of therapeutic escape, because cells in plastic states may be less proliferative, less immunogenic, more metabolically flexible, and more capable of surviving treatment. Importantly, phenotypic plasticity is often heterogeneous across tumors and even within individual lesions. LR signaling provides a plausible mechanism for this heterogeneity by allowing local immune context to shape tumor cell state in a niche-dependent manner. The fourth and perhaps most clinically consequential output is therapeutic resistance. Resistance can emerge against virtually all treatment modalities, including chemotherapy, targeted therapy, radiotherapy, endocrine therapy, and immune checkpoint blockade. Although the proximal mechanisms differ, many converge on similar adaptive programs: enhanced stress tolerance, suppression of cell death, maintenance of redox balance, altered nutrient utilization, and immune escape. LR signaling can contribute to all of these processes. For instance, inflammatory or chemokine-mediated receptor activation may promote glycolysis and anti-apoptotic signaling during chemotherapy; checkpoint-related signaling may coordinate immune exclusion and metabolic adaptation during immunotherapy; and macrophage- or myeloid-derived ligands may sustain survival pathways that undermine targeted inhibition (60). In this way, immune–tumor LR axes serve not only as modulators of tumor biology, but as active determinants of treatment response. These outputs are deeply interconnected. Metabolic rewiring may drive immune evasion; immune evasion may permit persistence of plastic subclones; plasticity may increase resistance to therapy; and therapy itself may amplify the microenvironmental conditions that activate LR signaling (61–64). This interconnectedness argues against studying any one of these phenotypes in isolation. Instead, it supports a model in which immune–tumor LR signaling acts as a higher-order regulatory layer that organizes multiple adaptive traits into coordinated resistance phenotypes. From a translational perspective, this framework is particularly useful because it identifies several levels of potential intervention. One can target the ligand, the receptor, downstream signaling nodes, the metabolic consequences of signaling, or the resistant cell state that emerges. Moreover, because many of these phenotypes are maintained by ongoing microenvironmental communication rather than permanent genetic alteration, they may be more therapeutically reversible than once assumed. Mapping the phenotypic consequences of LR signaling is therefore not only important for mechanistic understanding, but also for developing rational strategies to overcome resistance. Overall, immune–tumor ligand–receptor interactions should be regarded as organizing principles of tumor adaptation rather than isolated molecular events. Their downstream outputs—metabolic rewiring, immune evasion, phenotypic plasticity, and therapeutic resistance—collectively define the adaptive landscape in which cancer cells survive and evolve under therapeutic pressure. **Figure 1** illustrates immune–tumor ligand–receptor signaling as a central framework linking microenvironmental cues to tumor metabolic plasticity and adaptive resistance.

## 3 Metabolite-driven ligand–receptor axes linking tumor metabolism to immune escape

### 3.1 Lactate as a signaling metabolite rather than a passive by-product

Lactate has long been regarded as a terminal by-product of aerobic glycolysis and a biochemical hallmark of the Warburg phenotype. However, this traditional view is now clearly insufficient. In many cancers, lactate functions not merely as an indicator of altered metabolism, but as an active signaling metabolite that participates in bidirectional communication between tumor cells and the immune microenvironment (65–68). Its accumulation reflects high glycolytic flux, yet its biological effects extend far beyond energy metabolism, influencing receptor signaling, transcriptional regulation, immune suppression, and treatment responsiveness.

The signaling role of lactate is especially important in the context of immune–tumor communication. As glycolytic tumor cells export lactate into the extracellular milieu, they do more than acidify their surroundings; they create an information-rich metabolic niche that can be sensed by both tumor cells and infiltrating immune populations. Through receptor-mediated mechanisms and downstream signaling cascades, lactate can reshape tumor cell behavior in ways that reinforce malignant adaptation (69–71). This shift in perspective is conceptually important for understanding how metabolic reprogramming becomes functionally linked to immune escape. Rather than viewing metabolism and immunity as parallel processes, lactate reveals how they are mechanistically coupled.

One of the most striking features of lactate signaling is that it enables a metabolically advantaged tumor cell population to externalize its internal state and broadcast that state to neighboring cells. In other words, glycolytic activity becomes communicable. Tumor cells experiencing high anabolic demand, hypoxia, oncogenic signaling, or therapy-induced stress often increase lactate production as part of their adaptive metabolic response. Once secreted, lactate can signal that a glycolytic, stress-adapted state has emerged, and this signal can then trigger receptor-dependent programs that promote survival, checkpoint expression, immune suppression, or stromal remodeling. This makes lactate an unusually powerful link between cell-intrinsic metabolism and cell-extrinsic microenvironmental control. Lactate signaling is also important because it helps explain why metabolic adaptation often propagates beyond the individual tumor cell. A tumor region enriched in highly glycolytic cells may establish a lactate-rich microenvironment that affects less glycolytic tumor cells, myeloid populations, T cells, endothelial cells, and fibroblasts (72). In this way, lactate contributes to a field effect in which local metabolic reprogramming is converted into broader ecological change. Such metabolic field effects may be especially relevant under therapeutic pressure, when residual tumor cells rely heavily on supportive niches to endure cytotoxic, targeted, or immune-mediated attack.

Importantly, lactate signaling may influence checkpoint expression, suppressive immune-cell recruitment, myeloid polarization, and feedback loops that support tumor adaptation. Thus, lactate can be viewed not only as a metabolic product but also as a context-dependent signal that links glycolytic rewiring to immune suppression. The reconceptualization of lactate as a signaling metabolite has broad implications for therapeutic resistance. If lactate is simply a by-product, then its relevance is largely descriptive. If, however, it operates as an extracellular ligand-like cue that reshapes tumor–immune interactions, then it becomes a mechanistic driver of resistance-supportive niches. This distinction matters, because it suggests that targeting lactate production, transport, sensing, or downstream signaling could disrupt not only tumor metabolism itself, but also the immunosuppressive communication circuits that arise from that metabolism. Therefore, lactate occupies a central position at the intersection of tumor metabolic reprogramming and immune escape. It exemplifies how metabolites can function as signaling entities that actively coordinate adaptive phenotypes across the tumor ecosystem, laying the foundation for more durable resistance states.

### 3.2 GPR81/HCAR1-dependent immunosuppressive niche formation

Among the receptor systems implicated in lactate-mediated communication, G protein-coupled lactate receptors such as GPR81, also known as HCAR1, have emerged as particularly important mediators of immune–tumor crosstalk (73). These receptors help explain how extracellular lactate can be translated into structured biological responses rather than remaining a diffuse metabolic correlate. Through receptor engagement, lactate gains access to intracellular signaling programs capable of altering checkpoint regulation, chemokine production, immune cell recruitment, and the architecture of immunosuppressive niches. In tumor cells, lactate receptor signaling can reinforce adaptive phenotypes that support immune evasion. Rather than merely responding to the energetic state of the cell, receptor-mediated lactate sensing allows tumor cells to interpret extracellular metabolic conditions as actionable signals. This is particularly relevant in tumors where local lactate concentrations reflect sustained glycolysis, hypoxia, or therapy-induced stress. Under such conditions, activation of GPR81/HCAR1-related pathways can support signaling networks associated with survival, transcriptional plasticity, and immunoregulatory output. As a result, a metabolically stressed microenvironment is not passively tolerated; it is actively converted into a pro-tumor communication platform.

One important consequence of lactate receptor signaling is the enhancement of tumor immune tolerance through chemokine-mediated recruitment of suppressive immune cell populations (74). Lactate-sensing pathways can influence the expression of chemotactic molecules that selectively favor regulatory T cells, myeloid-derived suppressor cells, or alternatively polarized myeloid populations. This changes the cellular composition of the tumor microenvironment in a way that reinforces immune suppression and weakens effective antitumor responses. Thus, receptor-mediated lactate sensing is not only a biochemical adaptation but also an ecological strategy through which tumors reshape their immune surroundings.

This process also helps explain the self-reinforcing nature of glycolytic tumors. High glycolysis increases lactate production; lactate engages receptors and promotes immunosuppressive signaling; immunosuppressive cells then further support tumor survival and adaptation, enabling the maintenance of glycolytic and therapy-resistant states. In this sense, lactate receptor signaling closes a feedback loop between metabolic activity and immune dysfunction. The resulting niche is not simply characterized by high lactate, but by a coordinated set of tumor and immune cell states that sustain one another. Another notable feature of GPR81/HCAR1-dependent signaling is that it provides selectivity within a broadly abnormal metabolic landscape. Many metabolites accumulate in tumors, but only some are coupled to dedicated receptor systems capable of organizing structured multicellular responses. Lactate receptor engagement therefore represents a mechanism by which a ubiquitous metabolic alteration is converted into a specific signaling axis (75–78). This specificity makes the lactate–receptor interface particularly attractive conceptually and therapeutically, because it narrows the broad phenomenon of tumor metabolic dysregulation into targetable communication modules.

Lactate receptor signaling may also contribute to the persistence of drug-tolerant and immunotherapy-refractory tumor populations. Tumor cells that survive initial treatment often reside in metabolically and immunologically abnormal niches where extracellular lactate remains elevated. In such settings, receptor-dependent sensing can help residual cells maintain transcriptional and metabolic programs compatible with persistence. Meanwhile, suppressive immune subsets recruited or stabilized within these niches reduce the likelihood of effective immune-mediated clearance. Thus, the lactate–GPR81/HCAR1 axis may act as both a survival cue and a niche-maintenance system for residual disease. More broadly, GPR81/HCAR1-centered signaling illustrates an emerging principle in cancer biology: metabolite receptors can function as sensors of microenvironmental fitness landscapes. By detecting extracellular lactate, tumor or immune cells gain information about hypoxia, nutrient competition, inflammatory burden, and local cellular activity. The response to that information then shapes whether the niche becomes permissive for immune surveillance or resistant to it. In many aggressive tumors, the balance appears to shift toward the latter. Accordingly, GPR81/HCAR1-dependent signaling should be understood as a major mechanism by which glycolytic metabolism is translated into immunosuppressive tissue organization. It links the biochemical output of tumor metabolism to the cellular composition and functional orientation of the immune microenvironment, thereby contributing directly to the establishment of resistance-supportive niches.

### 3.3 Lactate transport and immune resistance

The biological influence of lactate depends not only on its production and receptor sensing, but also on its efficient movement across cellular membranes. Lactate transporters, particularly members of the monocarboxylate transporter family, play a crucial role in determining whether glycolytic activity remains a cell-intrinsic metabolic feature or becomes a microenvironmental force capable of shaping immune behavior. In this regard, lactate transport should be considered a functional extension of In this regard, lactate transport should be considered a metabolic infrastructure that enables extracellular lactate accumulation and thereby shapes the context in which metabolite-associated receptor signaling, such as lactate–GPR81/HCAR1 signaling, can occur.

Tumor cells with high glycolytic flux must continuously export lactate to avoid intracellular acidification and maintain metabolic throughput. This export allows the consequences of tumor metabolism to spill into the extracellular space, where lactate can act on neighboring cells and receptors. Without efficient transport, the signaling reach of lactate would be limited. Transporters therefore perform more than a clearance function; they help construct an extracellular metabolic niche that can support, but should be distinguished from, receptor-mediated lactate sensing. In practical terms, they determine the intensity, spatial spread, and persistence of lactate-associated signaling within the tumor microenvironment. This has major implications for immune resistance. By sustaining high extracellular lactate levels, transporter activity contributes to acidification, nutrient competition, and a metabolic environment that may favor local immunosuppressive signaling (79). Cytotoxic T cells and natural killer cells are particularly vulnerable to such conditions, as lactate-rich acidic environments can impair proliferation, cytokine production, and effector function. Meanwhile, suppressive immune populations often remain more adaptable within these conditions or may even be favored by them. Thus, transporter-mediated lactate accumulation indirectly influences which immune subsets thrive and which are disabled within the tumor niche.

Lactate transport may also contribute to intratumoral heterogeneity by generating local lactate gradients around highly glycolytic tumor regions. These gradients may help define metabolically stressed and immune-suppressed territories, although their functional relevance requires spatial and experimental validation. Beyond immune suppression, lactate transport contributes to therapy resistance by stabilizing broader adaptive programs. Efficient lactate export supports continued glycolysis, preserves redox balance, facilitates metabolic flexibility, and prevents toxic intracellular stress. At the same time, the resulting extracellular lactate pool reinforces receptor-mediated signaling and suppressive niche formation. In this way, transporters support two dimensions of resistance-associated metabolism: they help maintain intracellular metabolic homeostasis while increasing extracellular lactate availability that may influence neighboring immune and tumor cells. This dual role makes them highly consequential nodes in the adaptive resistance network.

Lactate transport may be especially important during treatment, when tumor cells face abrupt energetic and oxidative challenges. Under such conditions, reliance on glycolysis and lactate export can increase, and the extracellular consequences of this metabolic dependence may become even more pronounced. Residual tumor cells capable of maintaining efficient transporter activity may therefore gain a selective advantage not only because they withstand metabolic stress better, but also because they help sustain an immune microenvironment unfavorable to their elimination. In this sense, transporters do not merely assist resistant cells; they help build the niche in which resistance is maintained (80). The close relationship between lactate transport and immune resistance also highlights a broader conceptual point: metabolic communication in cancer is infrastructure-dependent. Metabolites can only function as intercellular signals if they are produced, exported, sensed, and integrated. Transporters form a central part of that infrastructure. Their contribution to tumor biology should therefore be interpreted not solely through the lens of metabolism, but also through their role in enabling cell–cell communication across the tumor ecosystem.

Taken together, Lactate transport is a critical amplifier of extracellular lactate accumulation and may indirectly enhance metabolite-associated immune–tumor signaling. By facilitating extracellular lactate accumulation and shaping the spatial and functional properties of the tumor microenvironment, transporters may help link metabolic rewiring to immune resistance, although they should be distinguished from classical ligand–receptor signaling components. This makes them an integral component of the broader communication axis linking glycolytic adaptation to therapeutic failure.

### 3.4 Lactate-centered feedback loops linking metabolism, immune suppression, and therapy resistance

An important reason lactate-centered communication is so relevant to cancer progression is that it rarely operates as an isolated event. Instead, lactate participates in feedback circuits that connect tumor metabolism, immune suppression, and therapeutic adaptation into a self-reinforcing system. These circuits help explain why glycolytic, immunosuppressive, and resistant phenotypes frequently coexist and why they can become progressively stabilized over time. A typical feedback loop begins with tumor-intrinsic or therapy-induced metabolic stress that increases glycolysis and lactate production. Lactate is then exported and sensed within the microenvironment, where it promotes immune dysfunction, suppressive cell recruitment, checkpoint upregulation, or receptor-dependent signaling in tumor cells (81–83). These changes reduce antitumor immune pressure and support the survival of metabolically aggressive tumor populations. As these cells persist, they continue to produce high levels of lactate, thereby sustaining the same conditions that protected them in the first place. What initially begins as an adaptive metabolic response is thus converted into an ecologically stable resistant niche.

These feedback loops may be strengthened by additional layers of signaling. Lactate-associated immune suppression can coexist with cytokine-mediated activation of survival pathways, chemokine-directed recruitment of myeloid cells, and extracellular matrix remodeling that further restricts immune infiltration. In parallel, chronic exposure to lactate-rich conditions may promote transcriptional states associated with checkpoint ligand expression, stress tolerance, and phenotypic plasticity. The end result is a deeply integrated network in which metabolic and immunologic adaptations are no longer separable. Tumor cells do not simply evade immune attack while remaining metabolically altered; rather, their altered metabolism actively helps generate the immune context that allows evasion to persist.

**Figure 2:**
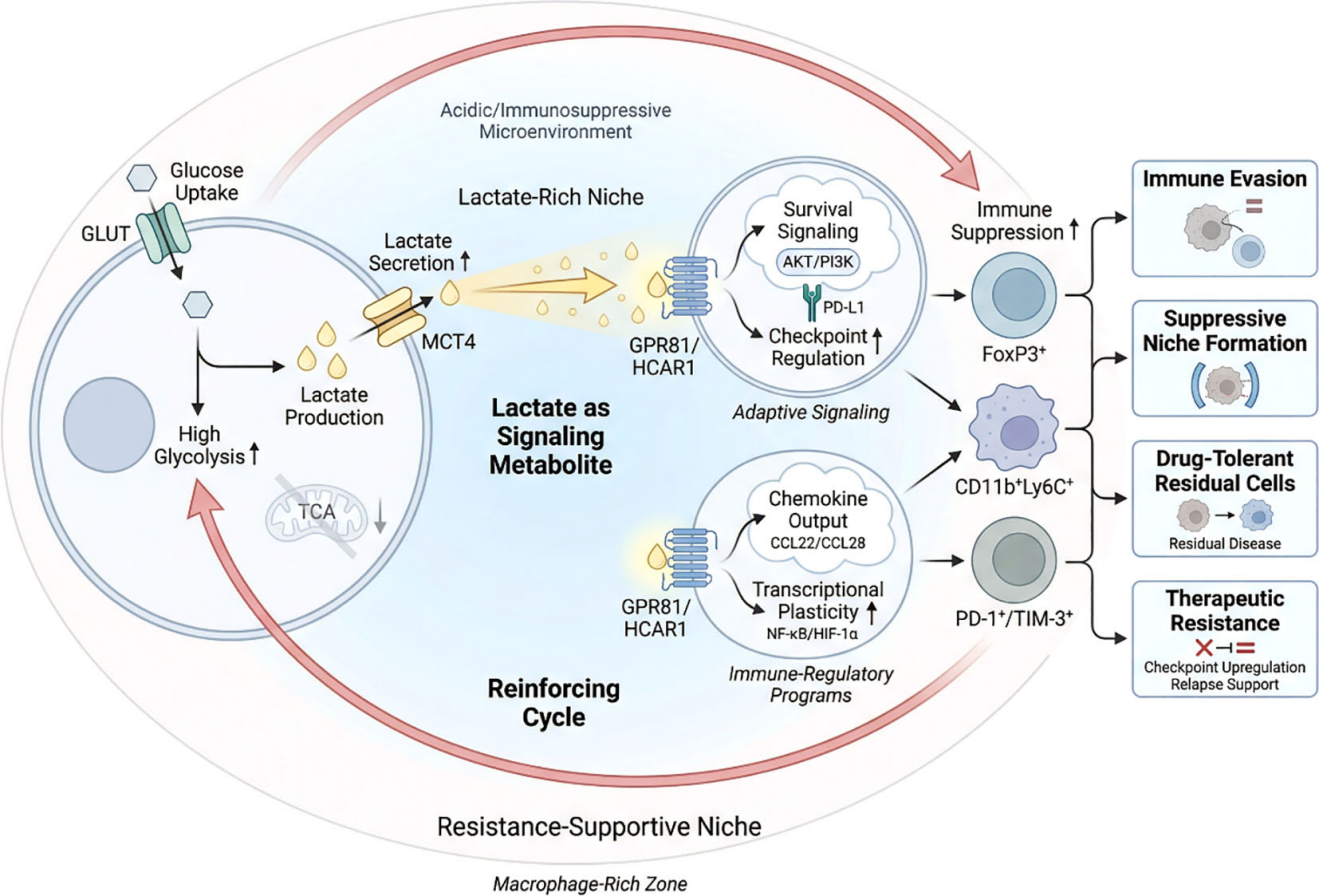
Lactate produced by glycolytic tumor cells is exported into the microenvironment, where it functions as a signaling metabolite rather than a passive by-product. Through GPR81/HCAR1-dependent sensing and related signaling programs, extracellular lactate promotes immune suppression, suppressive niche formation, residual tumor cell persistence, and therapeutic resistance.

This framework is especially relevant for understanding minimal residual disease and treatment relapse. Cells that survive initial therapy often occupy protective niches enriched in abnormal metabolites and suppressive immune populations. Lactate-centered signaling may contribute disproportionately to the maintenance of such niches because it links survival metabolism to local immune dysfunction. If these feedback systems remain intact after therapy, they can create a permissive environment for regrowth even when the bulk tumor burden has been substantially reduced. Accordingly, Lactate-centered axes may be viewed as dynamic organizing systems that contribute to the resistant tumor microenvironment. Their importance lies not only in the direct effects of lactate itself, but in their capacity to recruit, amplify, and stabilize other resistance-promoting processes (84, 85). This makes them particularly relevant to combination therapy design. Interventions aimed at lactate production, export, receptor sensing, or downstream signaling may have broader consequences than expected, precisely because they disrupt a feedback architecture rather than a single linear pathway. Overall, metabolite-driven ligand–receptor signaling centered on lactate provides one of the clearest examples of how tumor metabolism can be externalized into microenvironmental control. By linking glycolytic adaptation to immunosuppressive niche formation and therapy resistance, lactate-centered LR circuits may provide a conceptual bridge between metabolic rewiring and treatment failure in specific tumor contexts. **Figure 2** illustrates how lactate-centered ligand–receptor signaling converts tumor metabolic rewiring into microenvironmental control. It highlights a self-reinforcing circuit linking glycolysis, lactate export, immune suppression, and resistance-supportive niche stabilization.

## 4 Immune-derived cytokine and chemokine axes reprogram tumor cell metabolism

### 4.1 Chemokine receptor signaling and glycolytic adaptation

Chemokines have traditionally been studied as regulators of leukocyte trafficking, immune cell positioning, and inflammatory cell recruitment. In cancer, however, their function extends well beyond immune cell migration. A growing body of evidence indicates that Chemokine signaling may act directly on tumor cells to influence intracellular metabolism, thereby linking inflammatory communication to adaptive survival under therapeutic pressure. This conceptual expansion is important because it reframes chemokines from microenvironmental “recruitment cues” into active determinants of tumor cell metabolic state. Among the best examples of this principle is the CCL2-centered axis. CCL2 is widely recognized for its role in monocyte recruitment and myeloid enrichment, but it can also function as a tumor-directed signal that promotes metabolic and stress-adaptive programs (86, 87). In glioma cells, CCL2 has been shown to activate AKT signaling, enhance glycolytic activity, and promote chemoresistance, directly illustrating how an inflammatory ligand can couple receptor engagement to tumor metabolic adaptation and treatment tolerance (88). This is mechanistically important because AKT sits at a central node linking receptor signaling to glucose uptake, glycolytic enzyme activity, anabolic metabolism, and anti-apoptotic survival.

The significance of chemokine-driven glycolytic adaptation lies in its ability to provide tumor cells with a rapidly deployable resistance strategy. Glycolysis not only supports ATP generation under fluctuating oxygen conditions, but also supplies biosynthetic intermediates, sustains redox balance, and facilitates survival in stressed or damaged tissue. When chemokine signaling enhances glycolytic flux, tumor cells become better equipped to withstand cytotoxic therapy and persist in hostile microenvironments. Thus, the CCL2 axis exemplifies how inflammatory signaling can induce a metabolic state that is inherently compatible with therapy resistance (89–91). Chemokine receptor signaling also offers a plausible explanation for the spatial and temporal heterogeneity of tumor metabolism. Chemokine production is not uniformly distributed across the tumor microenvironment; rather, it varies according to myeloid cell density, tissue damage, vascular permeability, hypoxia, and treatment exposure. Consequently, tumor cells located near chemokine-rich immune niches may adopt more glycolytic and drug-tolerant phenotypes than cells in less inflamed regions. This helps explain why therapy-resistant subpopulations often emerge in discrete ecological niches rather than uniformly across the tumor mass.

Beyond CCL2, this framework likely applies to broader chemokine–receptor systems. Although the downstream details may differ, many chemokine receptors converge on signaling pathways such as AKT, ERK, STAT3, and NF-κB, all of which are capable of driving nutrient uptake, glycolytic reprogramming, mitochondrial adaptation, and survival signaling. In this sense, chemokine signaling should be viewed not only as a component of immune cell recruitment but also as a flexible upstream control layer for tumor metabolic plasticity. Importantly, chemokine-driven metabolic adaptation may also alter the tumor’s immune phenotype. Enhanced glycolysis frequently leads to increased lactate accumulation, acidification, and immune dysfunction, thereby extending the impact of chemokine signaling beyond tumor cell metabolism itself. A signal that initially promotes glycolysis in tumor cells may therefore ultimately reshape the entire microenvironment into one that is more permissive for persistence and less favorable to immune clearance. This provides a mechanistic bridge between inflammatory ligand exposure, metabolic rewiring, and immune escape.

Taken together, chemokine receptor signaling represents an important route through which immune-derived cues directly reprogram tumor metabolism. By promoting glycolytic adaptation and related stress-survival programs, these axes enable tumor cells to translate inflammatory pressure into treatment tolerance, making them central components of the broader network linking the immune microenvironment to cancer therapy resistance.

### 4.2 Macrophage-derived inflammatory signals and tumor metabolic survival programs

Among immune populations in the tumor microenvironment, macrophages are particularly influential regulators of tumor adaptation. Tumor-associated macrophages (TAMs) do not merely support progression through generalized immunosuppression; they also deliver highly instructive signals that shape tumor cell survival, plasticity, and metabolic behavior. Through the release of cytokines, chemokines, lipids, growth factors, and matrix-remodeling mediators, TAMs help create a tissue environment in which malignant cells can endure therapeutic stress and sustain growth despite immune and pharmacologic challenge. The interaction between macrophages and tumor cells is often bidirectional and functionally intimate. In small-cell lung cancer, direct cell–cell interaction between TAMs and tumor cells has been reported to promote tumor progression via STAT3 activation, underscoring how macrophage-derived signals can engage receptor-linked pathways in tumor cells and drive adaptive phenotypes (92). Although this study is not framed exclusively in metabolic terms, it is highly relevant to the present topic because STAT3 is one of the most important signaling hubs connecting inflammatory cues to metabolic survival programs, including glycolysis, mitochondrial fitness, redox adaptation, and anabolic support.

Macrophage-derived IL-6 is another especially important example. In breast cancer, glutathione S-transferase P1-mediated IL-6 production in TAMs has been shown to augment drug resistance in MCF-7 cells, providing direct evidence that a macrophage-derived inflammatory ligand can reinforce tumor survival under therapy (93). This finding is significant not only because it links TAM signaling to resistance, but also because IL-6/IL-6R signaling is deeply integrated with JAK/STAT3-dependent metabolic control. IL-6-driven signaling can enhance glycolytic enzyme expression, support anti-apoptotic programs, and favor cellular states that withstand chemotherapy-induced stress. Macrophage-derived inflammatory signaling may sustain resistant tumor states because it can be persistent, adaptable, and spatially organized within chronically inflamed, hypoxic, or therapy-exposed niches. In this way, TAM-derived ligands may provide external support for drug-tolerant metabolic and survival programs.

These survival programs are often metabolically encoded. Signals such as IL-6, CCL2, and other macrophage-associated mediators frequently converge on STAT3, AKT, and NF-κB, which together regulate glucose metabolism, mitochondrial resilience, biosynthetic flux, and antioxidant defenses. Such pathways can increase metabolic flexibility, allowing tumor cells to withstand nutrient limitation, oxidative damage, and cytotoxic exposure more effectively. In practice, this means that macrophage-derived signaling does not simply help tumor cells “survive” in an abstract sense; it helps them survive by maintaining the metabolic systems required for persistence (94–97). Because macrophage density and activation state vary across tumor regions, TAM-derived signals may contribute to spatial heterogeneity in drug-tolerant metabolic states. This spatial logic aligns with the broader idea that resistant states emerge within specialized microenvironmental territories rather than across the entire tumor in a homogeneous manner. Macrophage-derived signals may also cooperate with metabolite-driven pathways discussed earlier. A tumor cell exposed simultaneously to inflammatory cytokines and high extracellular lactate may receive both receptor-mediated survival instructions and metabolic support for immune evasion. These overlapping inputs can generate robust adaptive programs that are more stable than those driven by either signal alone. Thus, TAMs should not be considered isolated contributors to resistance, but key nodes in a multilayered communication network that integrates inflammatory signaling with metabolic rewiring.

Overall, macrophage-derived inflammatory signals represent a major class of immune-derived LR inputs that sustain tumor metabolic survival programs. By providing continuous, spatially organized, and pathway-convergent cues, TAMs help convert transient therapy-induced stress responses into stable resistance-supportive states.

### 4.3 Convergent signaling nodes linking inflammatory LR axes to metabolic adaptation

Although immune-derived cytokines and chemokines are diverse in origin and molecular identity, many of their effects on tumor metabolism converge on a limited set of intracellular signaling nodes. This convergence is one reason LR-mediated inflammatory communication is so effective in promoting adaptation: distinct extracellular cues can produce overlapping metabolic outcomes by funneling through shared downstream regulators. Among the most important of these are AKT, STAT3, and HIF-1α, with additional contributions from NF-κB, mTOR, and related transcriptional and metabolic effectors.

AKT is a prototypic metabolic integrator downstream of receptor signaling. Once activated by chemokine or cytokine-associated pathways, AKT can increase glucose uptake, stimulate glycolytic flux, support lipid synthesis, enhance mTOR-dependent biosynthesis, and suppress apoptotic signaling (98–100). In the context of inflammatory LR axes, AKT provides a rapid route through which extracellular immune cues are translated into cell-autonomous metabolic resilience. The study showing that CCL2 activates AKT signaling to promote glycolysis and chemoresistance in glioma cells is therefore conceptually important not only for CCL2 biology, but also for demonstrating how receptor-linked inflammatory signaling engages a central metabolic switch. STAT3 is another major convergence point. It has long been recognized as a transcription factor involved in inflammation, survival, stemness, and immune regulation, but its role in metabolic control is equally significant. STAT3 can support glycolysis, mitochondrial function, antioxidant defense, and biosynthetic programs that enable tumor persistence under stress (101–103). The observation that TAM–small-cell lung cancer interaction promotes tumor progression through STAT3 activation, together with evidence that TAM-derived IL-6 augments drug resistance, reinforces the view that STAT3 is a principal downstream effector through which macrophage-derived ligands shape tumor adaptation. In resistant tumors, STAT3 often functions less as a single-pathway endpoint and more as a transcriptional hub that consolidates multiple inflammatory inputs into a coordinated survival phenotype.

HIF-1α represents a third crucial node, particularly in regions where inflammation, hypoxia, and high glycolytic flux intersect. Even when not directly activated by oxygen deprivation alone, HIF-1α can be stabilized or functionally reinforced by inflammatory receptor signaling (104). Once engaged, it promotes glucose transporter expression, glycolytic enzyme induction, lactate production, and acid-resistant adaptation. This is especially relevant because HIF-1α can connect cytokine- and chemokine-driven signaling to the lactate-centered feedback circuits discussed in the previous section. In this way, inflammatory LR axes do not simply activate metabolism; they may help lock tumor cells into glycolysis-dominant, immunosuppressive states. These signaling nodes rarely operate independently. AKT, STAT3, HIF-1α, NF-κB, and mTOR form interconnected networks that integrate diverse LR inputs into overlapping metabolic and survival programs. This convergence may explain why single-axis blockade often produces incomplete responses and why combined targeting of upstream receptors and downstream metabolic dependencies may be required. The existence of these common downstream hubs has two important implications. First, it explains how tumors exposed to different immune ligands can nonetheless arrive at similar resistant metabolic states. Distinct upstream LR axes may converge on shared intracellular circuitry, producing parallel outputs such as glycolysis enhancement, redox protection, and anti-apoptotic signaling. Second, it identifies potential intervention points. While targeting every ligand individually may be impractical in highly heterogeneous tumors, disrupting shared downstream metabolic regulators or combining receptor blockade with metabolic therapy may prove more effective.

Thus, the study of immune-derived cytokine and chemokine axes should not end with ligand identification. Their full biological meaning emerges when they are mapped onto the intracellular hubs that actually execute metabolic adaptation. AKT, STAT3, and HIF-1α are especially important in this regard, because they convert inflammatory LR communication into durable changes in tumor cell metabolism, phenotypic plasticity, and therapy response.

### 4.4 Inflammatory LR signaling as a bridge between metabolic rewiring and therapeutic resistance

A central theme emerging from the studies above is that inflammatory LR signaling does not merely accompany therapeutic resistance; it actively structures the metabolic conditions under which resistance develops. By delivering external survival cues that reprogram glucose use, biosynthesis, redox balance, and stress signaling, immune-derived cytokines and chemokines help tumor cells adapt before, during, and after treatment exposure. This makes inflammatory communication a mechanistic bridge between microenvironmental pressure and resistant tumor metabolism. One important feature of this bridge is timing. Inflammatory signaling can act early, even before stable genetic resistance has emerged. Cytokines and chemokines released by macrophages and other immune cells may prime tumor cells into drug-tolerant metabolic states that allow them to survive the initial therapeutic insult. Cells that endure this early window of treatment can later accumulate additional adaptive changes, but their persistence may depend first on receptor-mediated microenvironmental support. In this sense, inflammatory LR axes may function as facilitators of resistance initiation rather than merely markers of established relapse.

**Figure 3:**
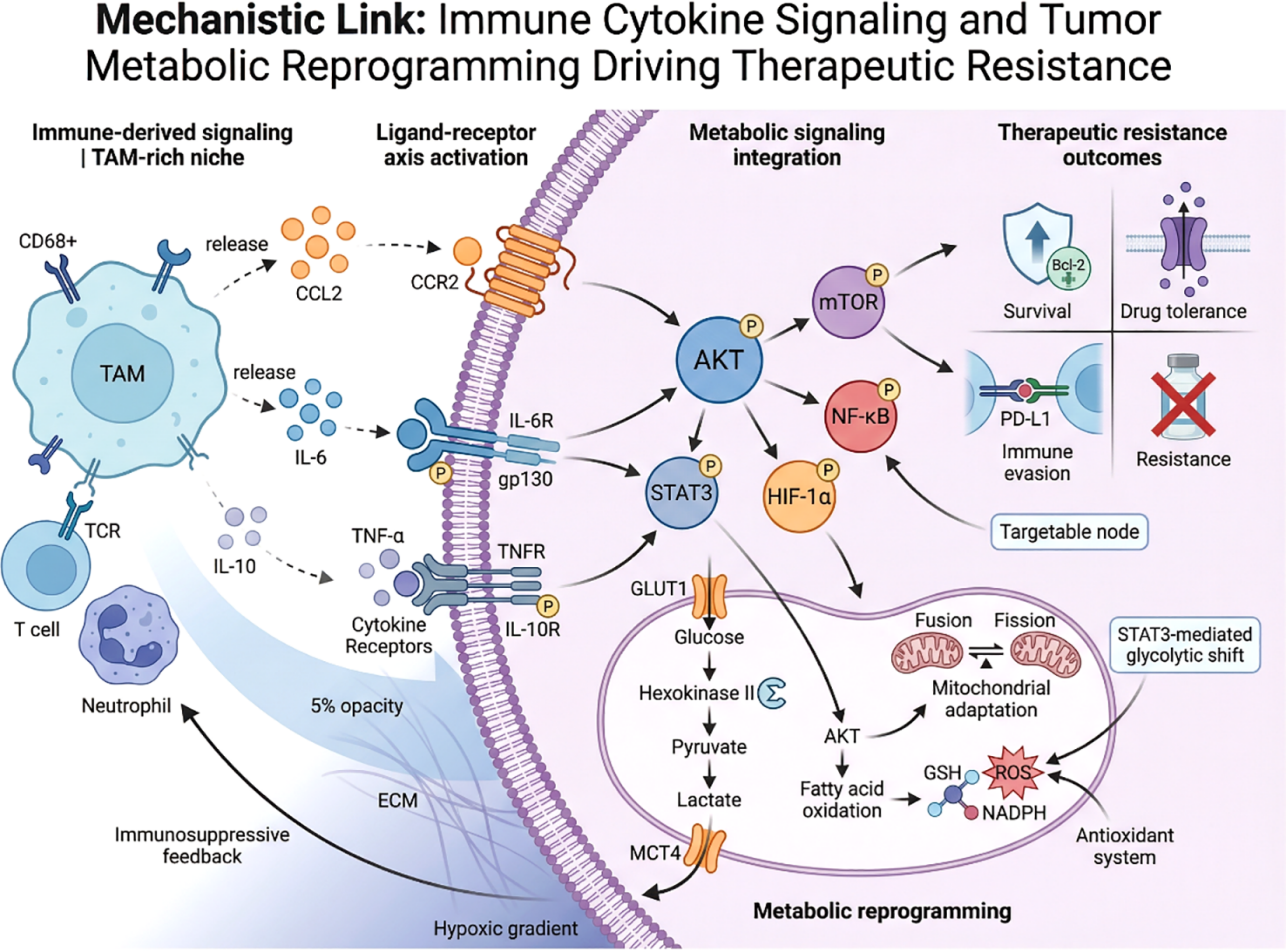
Immune-derived cytokines and chemokines, particularly from TAMs, engage tumor-cell receptors and converge on AKT, STAT3, HIF-1α, and related signaling hubs. These pathways promote glycolytic adaptation, metabolic plasticity, redox support, and survival signaling, thereby driving immune evasion, drug-tolerant states, and therapeutic resistance.

A second feature is reversibility. Because inflammatory signals are extrinsic, the metabolic states they sustain may be at least partially reversible if the supporting LR circuits are interrupted (105, 106). This distinguishes them from fixed genetic lesions and raises important therapeutic possibilities. A tumor cell maintained in a glycolytic, anti-apoptotic, or immune-evasive state by chronic cytokine exposure may become more vulnerable if the relevant ligand, receptor, or downstream pathway is blocked. Such reversibility is particularly relevant in combination therapy strategies aimed at collapsing residual disease niches rather than simply shrinking proliferative tumor bulk. A third feature is ecological amplification. Once inflammatory LR signaling has induced metabolic rewiring, the metabolic consequences of that rewiring can further reshape the immune context. Increased lactate production, oxidative stress adaptation, or altered nutrient competition may suppress effector immunity and favor myeloid persistence, thereby reinforcing the same inflammatory environment that supported resistance in the first place. This ecological amplification means that cytokine- and chemokine-driven metabolic adaptation is not a one-step process, but part of a recursive system that can become progressively self-sustaining. Overall, immune-derived cytokine and chemokine axes should be viewed as central regulators of tumor metabolic survival under therapeutic stress. They connect inflammation to tumor cell-intrinsic metabolic rewiring, and they translate microenvironmental signaling into phenotypes that ultimately determine treatment outcome. Understanding these pathways is therefore essential for any integrated model of how immune–tumor communication drives therapeutic resistance. **Figure 3** illustrates a proposed model in which immune-derived cytokine and chemokine signals may reprogram tumor-cell metabolism through convergent receptor-linked pathways. It highlights a resistance-supportive circuit in which inflammatory signaling may promote glycolytic adaptation, survival fitness, and persistent therapy tolerance. As summarized in **Table 1**, immune–tumor ligand–receptor axes converge on shared signaling nodes such as AKT, STAT3, and checkpoint-associated pathways to reprogram glycolysis, lipid metabolism, and suppressive niche formation. Representative original studies support lactate/GPR81 signaling, CCL2-driven glycolytic adaptation, TAM-derived IL-6 signaling, and PD-L1-associated metabolic rewiring as key mechanisms underlying this process (107).

**Table 1:** Representative immune–tumor ligand–receptor axes that drive metabolic reprogramming in cancer.

Immune/tumor-derived signal	Receptor or signaling interface	Major downstream pathway(s)	Metabolic consequence in tumor or niche	Biological outcome
Lactate	GPR81/HCAR1	TAZ-associated signaling, immunoregulatory transcriptional programs	Reinforced glycolytic niche, lactate-centered signaling, altered chemokine output	PD-L1 upregulation, immune suppression, Treg/MDSC recruitment
CCL2	CCR2-associated signaling	AKT	Enhanced glycolysis and metabolic stress adaptation	Chemoresistance, tumor survival
IL-6 from TAMs	IL-6R	JAK/STAT3	Increased glycolytic and survival-associated metabolic programs	Drug resistance, persistence under therapy
TAM–tumor contact-dependent inflammatory signaling	Multiple receptor-linked pathways	STAT3	Metabolic survival support and stress adaptation	Tumor progression, resistance-prone phenotype
PD-L1-associated signaling	PD-1/PD-L1-related checkpoint interface and tumor-intrinsic signaling	EGFR/ITGB4/SREBP1c, other survival pathways	Lipid metabolic reprogramming, glucose/fatty acid adaptation	Immune evasion, therapy tolerance
HK2-driven glycolytic state	Checkpoint-linked regulatory interface	Glycolysis/PD-L1 coupling	Increased glycolytic flux with checkpoint reinforcement	Immune escape, resistant phenotype
PKM2-linked glycolytic signaling	Checkpoint regulatory interface	Transcriptional/metabolic checkpoint control	Glycolysis-associated checkpoint expression	Tumor immune evasion

## 5 Immune checkpoint-associated ligand–receptor axes as regulators of tumor metabolism

### 5.1 PD-L1 as more than an immune checkpoint ligand

Programmed death-ligand 1 (PD-L1) has classically been understood as an immunoregulatory ligand that engages PD-1 on T cells to suppress antitumor immunity. Within this conventional framework, PD-L1 is primarily viewed as an immune-evasion molecule whose biological significance lies in dampening effector lymphocyte activation, cytokine production, and cytotoxic function. This view remains fundamentally important, but it is no longer sufficient. Increasing evidence suggests that PD-L1 may also exert tumor-intrinsic functions beyond immune suppression, including potential roles in survival signaling, phenotypic plasticity, and metabolic adaptation (108). Experimental studies in glioblastoma, breast cancer, liver cancer, and acute myeloid leukemia collectively support the idea that PD-L1 may be embedded in broader signaling networks associated with metabolism and tumor persistence.

This expanded view is relevant to immune–tumor communication but requires conceptual distinction. Canonically, PD-L1 functions as a checkpoint ligand in the PD-L1–PD-1 axis to regulate immune-cell behavior. In tumor cells, however, PD-L1 may also participate in tumor-intrinsic or membrane-proximal signaling programs that influence survival and metabolism; these functions are checkpoint-associated but are not necessarily equivalent to classical ligand–receptor signaling (109, 110). Its expression is dynamically shaped by metabolic state, and once expressed, it may also participate in intracellular or membrane-proximal signaling programs that feed back on tumor biology. In this way, PD-L1 occupies a strategic position at the interface of immune regulation and tumor cell adaptation. Rather than simply indicating that a tumor is “immune cold” or suppressive, PD-L1 may signify the presence of a broader adaptive state in which inflammatory pressure, metabolic rewiring, and therapeutic resistance are functionally connected. The conceptual importance of PD-L1 lies in its bidirectional relevance (111). On the one hand, metabolic and inflammatory signaling can drive PD-L1 upregulation, thereby converting tumor metabolic plasticity into an immune-evasive phenotype. On the other hand, tumor-cell PD-L1 has been linked to survival and metabolic programs, suggesting that checkpoint-associated molecules may also participate in cell-intrinsic adaptive signaling beyond their canonical ligand function. This bidirectionality makes checkpoint-associated LR axes especially powerful drivers of persistent resistant states. A tumor cell that upregulates PD-L1 in response to metabolic stress may simultaneously suppress immune clearance and strengthen its own metabolic fitness, creating a more robust adaptive phenotype than either process alone would provide.

Another reason to consider PD-L1 beyond its canonical immune function is that its effects appear to extend across multiple metabolic dimensions. Available evidence links PD-L1 not only to glycolytic regulation but also to lipid metabolic remodeling and broader survival-associated nutrient programs (112). This suggests that PD-L1 is part of a larger network of metabolic coordination rather than a narrow checkpoint endpoint. In resistant tumors, such coordination may be particularly advantageous, as it enables malignant cells to align immune evasion with the energetic and biosynthetic demands of therapy survival. This shift in perspective also has translational implications. If PD-L1 functions only as a T-cell inhibitory ligand, then anti-PD-1/PD-L1 therapy is interpreted mainly as an immunologic intervention. If, however, PD-L1 also supports tumor-intrinsic metabolic fitness, then checkpoint blockade may have broader biologic consequences, and resistance to checkpoint therapy may involve not only immune dysfunction but also persistent metabolic adaptation within tumor cells. This possibility helps explain why some tumors remain refractory to checkpoint blockade despite apparently adequate target expression and why PD-L1-high states may correlate with aggressive metabolic phenotypes.

Thus, PD-L1 should be viewed as more than a surface checkpoint ligand. It is increasingly recognized as a component of adaptive tumor circuitry that links extracellular immune pressure to intracellular metabolic and survival programs. Understanding this expanded role is essential for explaining how checkpoint-associated LR axes participate in the development of therapeutic resistance.

### 5.2 Glycolytic control of PD-L1 expression

One of the clearest demonstrations of the interface between tumor metabolism and checkpoint signaling is the regulation of PD-L1 by glycolytic machinery. Several original studies have shown that glycolytic enzymes are not merely metabolic executors but active determinants of checkpoint expression. In human glioblastoma cells, aerobic glycolysis was shown to promote tumor immune evasion through HK2-mediated PD-L1 upregulation, directly linking glucose metabolism to checkpoint control (113). A related study in breast cancer further demonstrated that HK2 promotes PD-L1 expression and immune evasion, indicating that this connection is not restricted to a single tumor type (114).

These findings are important because they reposition glycolysis as an upstream regulator of immune phenotype rather than a separate hallmark of tumor growth. In this model, metabolic activation is not simply parallel to immune escape; it actively generates immune escape by increasing PD-L1 abundance. Such a link provides a mechanistic basis for the frequent coexistence of high glycolytic activity and poor immune responsiveness in aggressive tumors. It also suggests that tumor cells can translate nutrient-rich or stress-adapted metabolic states into membrane-level immune inhibitory signals, thereby converting metabolic fitness into a direct survival advantage under immune pressure. The significance of HK2 in this context extends beyond its canonical role in catalyzing the first committed step of glycolysis (115, 116). HK2 is strategically positioned at the interface of metabolism, mitochondrial regulation, and stress adaptation. Its association with PD-L1 expression implies that key metabolic enzymes may function as signaling-sensitive nodes that coordinate energetic status with immune signaling outputs. This is particularly relevant in therapy-exposed tumors, where high glycolytic flux may arise in response to hypoxia, oncogenic signaling, or treatment-induced stress. Under such conditions, HK2-linked checkpoint induction could help residual cells both survive metabolically and evade immune elimination.

A complementary line of evidence comes from pyruvate kinase M2 (PKM2). PKM2 has been reported to be required for PD-L1 expression in immune cells and tumors, providing early and influential evidence that a central glycolytic regulator can directly shape checkpoint biology (117). This observation broadens the conceptual framework beyond HK2 and suggests that checkpoint regulation may be embedded within glycolytic control at multiple levels. Because PKM2 also participates in transcriptional and signaling functions beyond its enzymatic role, its involvement further supports the idea that metabolic enzymes can serve as integrators of inflammatory, metabolic, and immune-regulatory signals. Collectively, these studies suggest that glycolysis and PD-L1 expression are connected through a regulatory architecture rather than a simple correlation. Tumor cells with heightened glycolytic activity may be especially well positioned to sustain PD-L1-dependent immune suppression, while PD-L1-high tumors may in turn occupy metabolic states optimized for persistence under treatment. Such a coupling would be particularly advantageous in hostile microenvironments, where the ability to simultaneously secure metabolic support and suppress immune attack could determine whether a cell survives therapeutic challenge. This framework has direct implications for resistance biology. It suggests that tumors may escape immune control not only because PD-L1 is induced by inflammatory cytokines, but also because altered metabolism actively reinforces checkpoint expression. In practical terms, this means that metabolic inhibitors targeting glycolytic nodes such as HK2 or PKM2 could potentially affect tumor immunity as well as bioenergetics. The checkpoint phenotype may therefore be, at least in part, metabolically conditioned. Recognizing this is essential for understanding why metabolic rewiring and immune resistance so often emerge together.

### 5.3 PD-L1-driven glucose and lipid metabolic rewiring

If glycolysis can regulate PD-L1 expression, an important related question is whether tumor-cell PD-L1 can also participate in metabolic regulation. Recent studies suggest that the answer is yes. This represents a conceptual advance because it expands PD-L1 from a downstream immune marker to a checkpoint-associated molecule that may participate in tumor metabolic control. In liver cancer, tumor cell-expressed PD-L1 has been reported to promote lipid metabolic remodeling via EGFR/ITGB4/SREBP1c signaling, supporting the idea that PD-L1 may participate in lipogenic adaptation in an immune cell-independent manner (118). This is a particularly powerful observation because it establishes a direct mechanistic link between a canonical checkpoint ligand and a central anabolic metabolic pathway.

The importance of this finding lies in the role of lipid metabolism in tumor persistence. Lipid synthesis and remodeling support membrane biogenesis, receptor organization, signal transduction, oxidative stress buffering, and energy storage, all of which are advantageous under therapy-induced stress. If PD-L1 promotes such processes, then checkpoint signaling may help tumors coordinate immune evasion with anabolic adaptation. This dual role would be especially relevant in treatment-refractory disease, where cells must maintain both survival competence and resistance to immune-mediated elimination. In this context, PD-L1-associated lipid remodeling can be interpreted as one mechanism through which checkpoint-related biology may become metabolically relevant within tumor cells.

Evidence from acute myeloid leukemia further strengthens the case for tumor-intrinsic metabolic actions of PD-L1. PD-L1 stimulation has been reported to promote proliferation and survival of leukemic cells by influencing glucose and fatty acid metabolism, suggesting that checkpoint-associated signaling can modulate multiple metabolic branches simultaneously (119). This is important because it indicates that PD-L1-linked metabolic effects are not confined to solid tumors or lipid metabolism alone. Rather, PD-L1 appears capable of contributing to a broader nutrient-adaptive state that supports malignant survival across disease contexts. These studies together support a bidirectional model of checkpoint–metabolism coupling. In one direction, metabolic activation promotes PD-L1 expression. In the other, PD-L1 reinforces metabolic programs that sustain tumor growth, survival, and stress resistance. Such bidirectionality greatly increases the stability of resistant phenotypes. A tumor cell with elevated glycolysis may induce PD-L1, and PD-L1 may then further support lipid or glucose-associated adaptive pathways, creating a circuit in which metabolic and immune-evasive traits become mutually reinforcing (120, 121). This kind of circuit is particularly relevant to long-term persistence, because it reduces the likelihood that disrupting one arm of the system alone will be sufficient to reverse resistance. The ability of PD-L1 to shape metabolism also challenges a purely immune-centric interpretation of checkpoint blockade. If PD-L1 has tumor-intrinsic metabolic roles, then anti-PD-L1 therapies may influence cancer biology through mechanisms beyond T-cell reactivation. Conversely, tumors resistant to checkpoint blockade may remain so not only because of defective immune reinvigoration but also because PD-L1-associated metabolic circuits continue to support survival. This interpretation may help explain why some PD-L1-expressing tumors exhibit aggressive, therapy-resistant phenotypes even when immune infiltration is present.

Overall, PD-L1-driven metabolic rewiring represents a crucial component of adaptive tumor biology. By influencing glucose and lipid metabolism, PD-L1 contributes to a phenotype in which immune checkpoint signaling and metabolic resilience are tightly coupled. This makes checkpoint-associated LR axes especially potent regulators of therapeutic outcome.

### 5.4 Checkpoint-linked metabolic adaptation and immune resistance

The studies discussed above converge on a larger principle: checkpoint-associated communication and checkpoint-molecule-associated tumor-intrinsic programs are not simply endpoints of immune escape, but may participate in tumor adaptation through both immune and metabolic mechanisms. When checkpoint signaling becomes metabolically integrated, tumors gain access to a multidimensional resistance program. They can suppress cytotoxic immune responses, sustain nutrient-adaptive states, preserve biosynthetic and redox fitness, and stabilize drug-tolerant phenotypes simultaneously. This makes checkpoint-linked metabolic adaptation a particularly effective engine of persistent disease. One important consequence of this integration is that immune resistance can become metabolically self-reinforcing. Glycolytic activation promotes PD-L1 expression, PD-L1 can then support additional metabolic rewiring, and the resulting state may favor lactate accumulation, immune dysfunction, and survival under therapy (122). This architecture is highly compatible with the development of refractory tumor niches. Once established, such niches may not depend on any single ligand or pathway but on the collective maintenance of checkpoint signaling, metabolic flexibility, and suppressive microenvironmental conditions. This may help explain why checkpoint resistance is often durable and why tumors can remain refractory even after partial immune reinvigoration.

Checkpoint-linked metabolic adaptation also provides a framework for understanding heterogeneity in immunotherapy response. Tumors with superficially similar PD-L1 expression may differ substantially in the metabolic context underlying that expression. In some cases, PD-L1 may reflect transient inflammatory induction; in others, it may be embedded in a stable glycolytic or lipogenic program that is much harder to reverse. This distinction is clinically meaningful because checkpoint blockade may be more effective when PD-L1 expression is not reinforced by deep metabolic adaptation. Thus, checkpoint status alone may be insufficient to predict response unless interpreted alongside the metabolic architecture of the tumor. From a therapeutic perspective, these insights support the rationale for combination strategies that target both checkpoint signaling and tumor metabolism. If glycolytic enzymes, lactate-associated pathways, or lipid metabolic regulators help sustain checkpoint-dependent resistance, then combining metabolic intervention with PD-1/PD-L1 blockade may disrupt the adaptive circuitry more effectively than either approach alone (123). This does not imply that all metabolically altered tumors will respond to such combinations, but it does suggest that checkpoint resistance is best understood as a systems problem involving immune communication and metabolic state rather than a single-axis defect.

**Figure 4:**
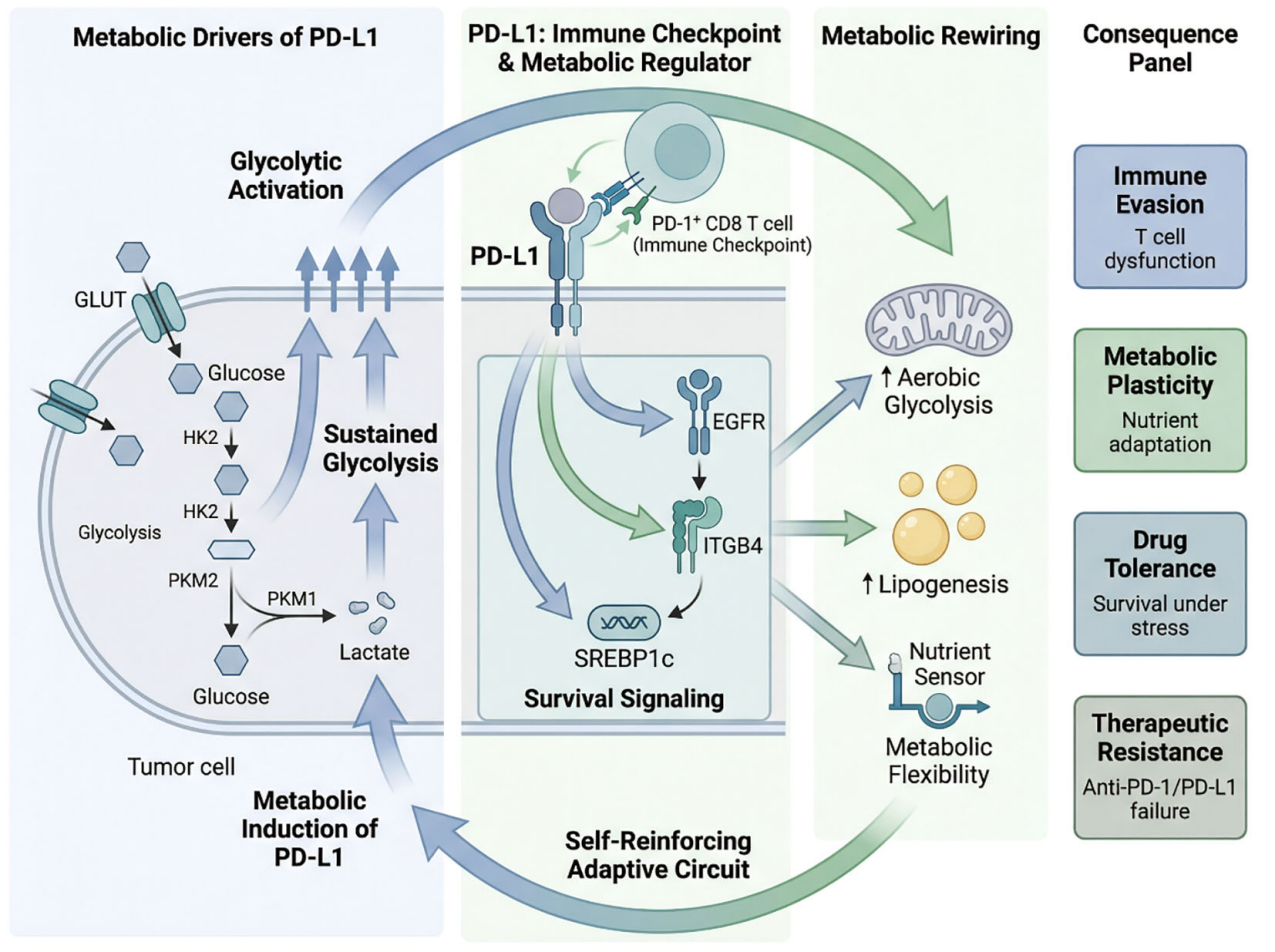
Glycolytic activation promotes PD-L1 expression, while PD-L1 further reinforces tumor-intrinsic glucose and lipid metabolic rewiring. This bidirectional checkpoint–metabolism circuit couples immune evasion to metabolic resilience, thereby stabilizing drug-tolerant states and persistent therapeutic resistance.

In summary, immune checkpoint-associated ligand–receptor axes and tumor-intrinsic checkpoint-molecule functions occupy important but distinct positions in the crosstalk between tumor immunity and metabolism. Canonical checkpoint LR signaling is important for suppressing antitumor immune responses, whereas tumor-intrinsic checkpoint-molecule functions may support metabolic programs associated with persistence under therapeutic stress. Recognizing this dual function is essential for a more complete understanding of cancer therapy resistance and provides a foundation for the next section, which examines how immune–tumor LR axes drive resistance across different treatment modalities more broadly. **Figure 4** illustrates the dual but distinct roles of PD-L1 as a canonical immune checkpoint ligand in the PD-L1–PD-1 axis and as a tumor-intrinsic checkpoint-associated molecule linked to metabolic regulation. It highlights a self-reinforcing circuit in which glycolytic activation induces PD-L1, and PD-L1 further promotes metabolic adaptation, immune evasion, and therapy resistance.

## 6 Immune–tumor LR axes in the acquisition of therapeutic resistance

### 6.1 Chemotherapy resistance

Chemotherapy remains a cornerstone of cancer treatment across both solid and hematologic malignancies, yet the emergence of resistance continues to limit its long-term benefit. Although chemotherapy resistance is often attributed to tumor-intrinsic mechanisms such as enhanced drug efflux, defective apoptosis, altered DNA damage repair, and epigenetic adaptation, these explanations do not fully account for the dynamic and microenvironment-dependent nature of treatment failure. Increasing evidence suggests that immune–tumor ligand–receptor communication plays an important role in establishing the metabolic and signaling conditions under which chemoresistant phenotypes arise and persist.

One major mechanism through which immune-derived LR axes promote chemotherapy resistance is the induction of metabolic states that buffer tumor cells against cytotoxic stress (124–126). Inflammatory chemokines and cytokines released by immune cells can activate receptor-linked pathways in tumor cells that favor glycolysis, anabolic support, redox homeostasis, and anti-apoptotic signaling. The CCL2-centered axis provides a representative example: by activating AKT signaling and promoting glycolysis, CCL2 enhances chemoresistance in glioma cells, illustrating how an inflammatory ligand can directly shift tumor metabolism toward a drug-tolerant state. In this context, glycolytic adaptation is not merely a metabolic accompaniment of resistance, but a functional mechanism that helps tumor cells survive chemotherapy-induced energetic and oxidative stress.

Macrophage-derived inflammatory signals are also central to this process. TAMs can serve as persistent sources of soluble ligands that support tumor cell survival during chemotherapy exposure. IL-6 is especially important because it links inflammatory communication to transcriptional programs governing metabolism, stress adaptation, and survival. Evidence that glutathione S-transferase P1-mediated IL-6 production in TAMs augments drug resistance in breast cancer cells supports the view that macrophage-derived cytokine signaling can actively maintain chemoresistant phenotypes (101). Such observations suggest that chemotherapy resistance should not be understood only as a property acquired by tumor cells, but also as a phenotype stabilized by surrounding immune niches. Checkpoint-associated LR axes may further reinforce chemotherapy resistance. In ovarian cancer, upregulation of PD-L1 in TAMs has been shown to affect chemotherapeutic response, highlighting an additional layer through which immune suppressive communication intersects with treatment outcome (127). This is conceptually important because it suggests that checkpoint signaling can influence not only immunotherapy responsiveness but also the tumor’s broader response to cytotoxic drugs. In such settings, chemotherapy resistance may be coupled to an immune-suppressive microenvironment that reduces immune-mediated clearance of damaged tumor cells and supports the survival of residual disease.

The contribution of immune–tumor LR axes to chemotherapy resistance is particularly relevant in the setting of residual tumor persistence. Cytotoxic therapy rarely eliminates all malignant cells simultaneously. Instead, a subset of cells survives and enters a stress-adapted state in which dependence on microenvironmental support may increase. Immune-derived ligands can help sustain this state by maintaining receptor-mediated survival signaling and metabolic flexibility. Under these conditions, resistant cells are not merely selected; they are actively supported. This distinction has major therapeutic implications because it implies that disrupting microenvironmental LR support could sensitize tumor cells that would otherwise remain drug tolerant. Another important aspect is spatial heterogeneity. Chemotherapy does not act uniformly across a tumor, and neither do immune signals. Regions enriched in macrophages, myeloid cells, inflammatory chemokines, or checkpoint-associated signaling may provide local sanctuaries where tumor cells are better able to endure treatment. Within these niches, ligand–receptor communication can preserve metabolic resilience and suppress apoptosis long enough for surviving cells to recover, adapt, and ultimately drive relapse. Thus, chemoresistance may emerge not simply from cell-autonomous Darwinian selection, but from ecological support systems embedded within the tumor microenvironment. Overall, chemotherapy resistance is increasingly understood as a phenotype shaped by the interaction between tumor cell stress responses and immune-derived signaling cues. Immune–tumor LR axes contribute to this process by promoting glycolytic adaptation, reinforcing survival pathways, stabilizing redox homeostasis, and sustaining residual disease niches. Appreciating this extrinsic dimension of chemoresistance is essential for developing strategies that go beyond targeting tumor cells alone.

### 6.2 Targeted therapy resistance

Targeted therapy resistance differs from chemotherapy resistance because it frequently emerges through pathway bypass, receptor rewiring, lineage plasticity, and adaptive survival signaling rather than through nonspecific cytotoxic stress alone (128, 129). Under selective pressure from kinase inhibitors or other targeted agents, tumor cells may restore downstream signaling through alternative receptor inputs supplied by immune or stromal cells. In this context, LR axes such as HGF–MET, EGF–EGFR, AXL-related signaling, TGF-β–TGFBR, and CXCL12–CXCR4 may reactivate convergent nodes including PI3K/AKT, MAPK/ERK, STAT3, and mTOR, thereby supporting survival despite inhibition of the original oncogenic driver (130–133). These compensatory LR cues can also reshape tumor metabolism. Depending on tumor type and targeted agent, bypass signaling may promote glycolytic switching, mitochondrial adaptation, lipid remodeling, amino acid utilization, redox buffering, EMT-like plasticity, or slow-cycling drug-tolerant states. TAMs, CAFs, endothelial cells, and therapy-remodeled stromal niches may provide cytokines, chemokines, and growth factors that reinforce these adaptive programs. For example, macrophage-derived inflammatory signals may support STAT3- or AKT-associated survival, whereas CAF-derived TGF-β or CXCL12 may contribute to stromal protection, invasive plasticity, and immune exclusion (134, 135). Thus, targeted therapy resistance is often shaped by compensatory receptor signaling and microenvironment-derived bypass cues together with metabolic flexibility. These mechanisms support rational combination strategies. Targeted agents may need to be paired with inhibitors of compensatory LR pathways, such as MET, AXL, CXCR4, TGF-β, STAT3, or PI3K/AKT/mTOR, or with interventions targeting lipid metabolism, mitochondrial adaptation, or redox defense. Such combinations should be guided by tumor type, dominant bypass pathway, stromal context, and longitudinal changes during therapy rather than applied uniformly.

### 6.3 Radiotherapy resistance

Radiotherapy resistance is strongly influenced by hypoxia, redox balance, DNA damage repair capacity, and immune remodeling after irradiation. Radiation can induce inflammatory and damage-associated signals that alter immune-cell recruitment and stromal organization (136, 137). In this setting, LR axes such as CSF1–CSF1R, CXCL12–CXCR4, TGF-β–TGFBR, and macrophage-derived cytokine signaling may contribute to TAM accumulation, immune suppression, tissue repair-like programs, and survival of irradiated tumor cells. For example, CSF1R blockade has been shown to suppress tumor-infiltrating myeloid cells and improve radiotherapy efficacy in prostate cancer (138), CXCL12 mediates glioblastoma resistance to radiotherapy in the subventricular zone (139), and TGF-β inhibition can abolish microenvironment-mediated radioresistance of glioblastoma-initiating cells (140). Hypoxic and lactate-rich regions may further reduce radiosensitivity by limiting oxygen-dependent DNA damage and promoting antioxidant adaptation, while radiation-induced lactate has been shown to enhance the immunosuppressive activity of MDSCs through a GPR81/mTOR/HIF-1α/STAT3 pathway in pancreatic cancer models (141). Thus, radiotherapy resistance is particularly linked to spatially organized hypoxic niches, myeloid recruitment, redox adaptation, and tissue-repair-associated LR signaling.

### 6.4 Resistance to immune checkpoint blockade

Immune checkpoint blockade (ICB) has transformed cancer therapy, yet primary and acquired resistance remain major clinical obstacles. Conventional explanations for ICB resistance often emphasize insufficient neoantigen burden, defective antigen presentation, T-cell exclusion, compensatory checkpoint pathways, or irreversible T-cell dysfunction. While these mechanisms are important, they do not fully capture how tumor cells adapt to immune pressure at the interface of metabolism and microenvironmental communication. Immune–tumor LR axes provide a useful framework for understanding this broader adaptive landscape because they integrate checkpoint signaling with inflammatory cues, myeloid reprogramming, metabolic remodeling, and tissue niche organization. A key feature of ICB resistance is that it is not solely a failure of T-cell activation. In many tumors, resistance reflects the persistence of a communication network that continually re-establishes suppressive conditions even in the presence of checkpoint blockade. Lactate-centered signaling, macrophage-derived inflammatory cues, and checkpoint-associated metabolic circuits can all contribute to this problem by maintaining an immune-excluded or immune-dysfunctional niche. Under such conditions, blocking PD-1 or PD-L1 may relieve one inhibitory interaction without dismantling the broader system that supports tumor survival and immune evasion.

Recent studies illustrate how defined LR circuits can actively promote resistance to checkpoint therapy. In bladder cancer, LRP1 has been shown to induce anti-PD-1 resistance by modulating the DLL4-NOTCH2-CCL2 axis and redirecting M2-like macrophage polarization (142). This is particularly relevant to the present review because it demonstrates that resistance to immunotherapy can be driven by a structured ligand–receptor communication axis rather than by a diffuse suppressive environment alone. The DLL4-NOTCH2-CCL2 network links receptor-mediated signaling to myeloid remodeling, thereby creating a tumor-supportive immune context that undermines checkpoint efficacy. Similarly, in non-small cell lung cancer, ALKBH5 has been reported to promote tumor progression and alter susceptibility to anti-PD-L1 therapy by modulating tumor–macrophage interactions (143). This finding reinforces the broader principle that tumor response to checkpoint blockade depends not only on target expression, but also on the quality of immune–tumor communication. Macrophage-centered LR signaling may determine whether the tumor microenvironment remains suppressive despite checkpoint inhibition, especially when these interactions are coupled to metabolic adaptation and survival signaling within tumor cells.

Another important consideration is that checkpoint resistance may be metabolically entrenched. As discussed in the previous section, glycolysis can promote PD-L1 expression, and PD-L1 itself can support tumor-intrinsic metabolic programs. This means that ICB-resistant tumors may harbor checkpoint-associated adaptive states that are maintained by persistent metabolic rewiring. Even if antibody-mediated blockade interrupts receptor engagement at the immune synapse, the tumor may remain in a metabolically robust, immune-evasive condition that continues to favor survival. In such cases, checkpoint resistance should be understood not simply as ineffective immune reactivation, but as the persistence of a tumor state in which immune escape and metabolic plasticity are already tightly coupled. Myeloid cell reprogramming is especially relevant here. Tumors resistant to ICB frequently contain macrophages, monocytes, and myeloid-derived suppressor cells that secrete chemokines, cytokines, and metabolites capable of dampening cytotoxic immunity and supporting tumor adaptation. LR axes that favor M2-like polarization, suppress antigen presentation, or recruit immunosuppressive populations can blunt the effect of checkpoint blockade even when effector T cells are present (144). Thus, resistance to ICB often reflects not a single failed interaction, but a microenvironmental circuit in which immune and metabolic suppression are co-maintained. These insights have important therapeutic implications. They suggest that successful reversal of ICB resistance may require more than targeting PD-1/PD-L1 alone. It may also be necessary to disrupt the LR networks that sustain suppressive myeloid niches, metabolic adaptation, or checkpoint-associated survival programs in tumor cells. In this regard, combining checkpoint blockade with interventions targeting chemokine signaling, macrophage polarization, lactate-associated pathways, or shared downstream metabolic nodes may offer a more effective strategy than checkpoint monotherapy.

In summary, resistance to immune checkpoint blockade is often driven by a complex LR-mediated ecosystem rather than by isolated defects in T-cell function. Immune–tumor communication networks involving macrophages, chemokines, checkpoint-associated signaling, and metabolic rewiring can preserve suppressive tumor states despite therapy. Understanding these axes is therefore essential for explaining why many tumors remain refractory to ICB and for designing strategies capable of overcoming that resistance.

### 6.5 Shared mechanisms of multi-therapy resistance

Although chemotherapy resistance and immunotherapy resistance are often studied as distinct clinical problems, they share a number of underlying biological mechanisms. From the perspective of immune–tumor ligand–receptor communication, this overlap is not surprising. Many LR axes do not act in a therapy-specific manner; rather, they induce adaptive tumor states that are broadly compatible with survival under multiple forms of stress. These shared states include sustained glycolysis, lipid remodeling, redox buffering, anti-apoptotic signaling, phenotypic plasticity, immune suppression, and supportive niche remodeling. As a result, the same microenvironmental communication circuits may contribute simultaneously to resistance against chemotherapy, targeted therapy, and immunotherapy.

One common mechanism is metabolic flexibility. Tumor cells that receive continuous receptor-mediated support from inflammatory cytokines, chemokines, or checkpoint-associated signaling are often better able to shift between metabolic programs in response to changing therapeutic conditions. Such cells may increase glycolysis during acute stress, rely on lipid metabolism during prolonged survival, or enhance antioxidant pathways to withstand oxidative damage (145, 146). This flexibility makes them less vulnerable to any single therapeutic insult. Rather than being specifically resistant to one treatment, they enter a generalized adaptive state that improves fitness across multiple challenges. A second shared mechanism is immune suppression coupled to residual disease survival. Treatments as different as cytotoxic chemotherapy and checkpoint blockade can fail when surviving tumor cells remain embedded in niches that prevent effective immune clearance. As discussed in Sections 3–5, lactate-centered signaling, TAM-derived cues, and checkpoint-associated programs may each contribute to residual disease niches. Here, we emphasize their shared consequence: metabolically and immunologically protected tumor-cell persistence after therapy.

A third shared mechanism is signaling redundancy and pathway convergence. Distinct ligands may activate overlapping downstream pathways such as AKT, STAT3, HIF-1α, NF-κB, or mTOR (147, 148). Once these hubs are engaged, tumor cells can acquire similar resistant phenotypes even when the initiating LR axis differs. This redundancy helps explain why blocking a single upstream ligand often yields incomplete or transient benefit. It also suggests that multi-therapy resistance may be driven less by any one molecule than by dense signaling architectures that stabilize adaptation across diverse contexts. Another shared feature is phenotypic plasticity. LR-associated communication may support quiescent, stem-like, mesenchymal, or stress-tolerant states that allow tumor cells to survive sequential therapeutic pressures. This concept links distinct resistance phenotypes without requiring repeated discussion of each upstream LR axis. These shared mechanisms argue strongly for an integrated therapeutic perspective. Rather than treating each resistance modality as biologically isolated, it may be more effective to identify the immune–tumor LR circuits that sustain adaptive survival across therapies. Such circuits may represent higher-order vulnerabilities. Targeting them could theoretically sensitize tumors to multiple treatments at once or prevent the sequential evolution of resistant states during therapy switching.

Overall, multi-therapy resistance reflects the capacity of tumors to organize broad adaptive programs in response to persistent microenvironmental pressure. Immune–tumor LR axes are central to this organization because they translate inflammatory, metabolic, and checkpoint-related signals into durable survival phenotypes. Recognizing the shared mechanisms across treatment modalities not only clarifies why resistance is so difficult to overcome, but also highlights why strategies aimed at disrupting communication networks may be especially valuable. As outlined in **Table 2**, immune–tumor LR circuits support resistance across treatment modalities by coupling inflammatory signaling to glycolytic adaptation, lipid remodeling, and suppressive niche maintenance. Original studies have linked these axes to chemoresistance, anti-PD-1 resistance, and persistent immune-evasive states.

**Table 2:** Immune–tumor ligand–receptor-mediated metabolic programs in therapeutic resistance.

LR axis/communication module	Dominant metabolic program	Type of resistance	Representative resistance mechanism	Potential intervention direction
Lactate–GPR81/HCAR1 axis	Glycolysis-high, lactate accumulation	Immune resistance/ICB resistance	Treg or MDSC recruitment, checkpoint reinforcement, suppressive niche formation	Lactate production blockade, lactate transport inhibition, receptor targeting
CCL2–CCR2 axis	AKT-driven glycolytic adaptation	Chemotherapy resistance	Enhanced glucose utilization and survival signaling under cytotoxic stress	CCR2/chemokine blockade plus chemotherapy/metabolic targeting
IL-6–IL-6R axis	STAT3-linked metabolic survival program	Chemotherapy resistance	Anti-apoptotic and stress-adaptive metabolic rewiring	IL-6/STAT3 inhibition plus cytotoxic therapy
TAM PD-L1–associated signaling	Checkpoint-linked metabolic adaptation	Chemotherapy resistance/immune escape	Reduced immune-mediated clearance of damaged tumor cells	TAM reprogramming plus checkpoint blockade or chemotherapy
PD-L1 tumor-intrinsic signaling	Lipid and glucose metabolic rewiring	Immune checkpoint blockade resistance/broad therapy tolerance	Persistent metabolic fitness despite immune pressure	PD-1/PD-L1 blockade plus metabolic inhibition
DLL4–NOTCH2–CCL2 axis	Myeloid-supported adaptive niche metabolism	Anti-PD-1 resistance	M2-like macrophage polarization and suppressive niche maintenance	Notch/chemokine/myeloid-targeted combination strategies
Tumor–macrophage interaction programs	Glycolytic, lipogenic, and stress-adaptive plasticity	Multi-therapy resistance	Persistent myeloid support for residual disease	Macrophage-targeted therapy plus standard-of-care treatment

### 6.6 Tumor-type-specific LR–metabolic resistance programs

Although immune–tumor ligand–receptor axes provide a useful framework for understanding adaptive resistance, these axes should not be interpreted as uniformly operating across all cancer types. Distinct tumors possess different tissue origins, immune ecosystems, metabolic dependencies, stromal architectures, and therapeutic selection pressures. Therefore, the dominant LR–metabolic resistance programs are highly context dependent.

In gliomas, the resistant microenvironment is frequently shaped by myeloid-cell dominance, limited cytotoxic T-cell infiltration, hypoxia, and lactate accumulation (149). In this setting, lactate-associated signaling, hypoxia-driven HIF-1α activation, CCL2-related myeloid recruitment, and macrophage-derived inflammatory cues may be particularly relevant to metabolic adaptation and therapeutic tolerance (88). These axes may contribute to glycolytic remodeling, immune exclusion, and survival of tumor cells in poorly perfused and immunologically suppressed niches (88). In hepatocellular carcinoma, LR–metabolic resistance mechanisms are strongly influenced by the immune-tolerant hepatic microenvironment, chronic inflammation, lipid metabolic rewiring, and checkpoint regulation (118, 150). PD-L1-associated signaling, IL-6/STAT3 activation, macrophage-derived chemokine axes, and growth factor receptor pathways may cooperate with lipid metabolism and inflammatory survival programs to support resistance to targeted therapy and immune checkpoint blockade (118). Thus, in HCC, LR-driven metabolic resistance is closely linked to liver-specific immune tolerance and inflammation-associated metabolic remodeling. In breast cancer, particularly triple-negative and therapy-refractory subtypes, TAMs, CAFs, lactate-rich niches, IL-6 signaling, EMT programs, and chemotherapy-induced inflammatory remodeling are central features of resistant ecosystems (93, 151, 152). TAM-derived IL-6, CCL2/CCR2-related communication, CAF-derived metabolic support, and lactate-mediated immune suppression may converge on STAT3, AKT, HIF-1α, and EMT-associated pathways, thereby promoting glycolytic adaptation, invasive plasticity, and chemotherapy resistance. In non-small cell lung cancer, resistance is often associated with immune checkpoint failure, CXCL/CXCR chemokine signaling, TAM-mediated suppression, T-cell exclusion, and therapy-induced inflammatory remodeling (107). In this context, PD-L1-centered checkpoint–metabolic coupling, CXCL12–CXCR4 or CXCL8–CXCR1/2 signaling, and macrophage-derived inflammatory axes may reinforce immune evasion and adaptive metabolic states that limit response to immune checkpoint blockade and targeted therapy. In gastric and colorectal cancers, stromal-rich and myeloid-enriched microenvironments are particularly important. CAF-derived TGF-β signaling, SPP1-positive macrophage-associated communication, chemokine-mediated myeloid recruitment, and metabolic barriers such as lactate accumulation, hypoxia, and nutrient competition may collectively promote immune exclusion, epithelial–mesenchymal plasticity, and therapeutic resistance (153, 154). These tumors therefore illustrate how LR–metabolic resistance can be organized by stromal–myeloid niches rather than by tumor cells alone.

Together, these examples indicate that immune–tumor LR axes act as a general organizing principle, but their dominant components, metabolic outputs, and resistance phenotypes are tumor-type specific. Future studies should therefore avoid treating LR interactions as pan-cancer signatures without functional validation. Instead, LR–metabolic axes should be interpreted in relation to tissue context, immune composition, spati.

### 6.7 A unifying view of LR-mediated therapeutic resistance

Taken together, the evidence reviewed in this section supports a unifying model in which immune–tumor LR axes function as adaptive control systems for therapeutic resistance. In this model, resistance is not simply the endpoint of tumor-intrinsic evolution, nor merely the consequence of a suppressive microenvironment. Instead, It may emerge from continuous signaling exchange between tumor cells and immune populations, with ligand–receptor communication contributing to the metabolic, transcriptional, and ecological states associated with tumor-cell survival under treatment.

**Figure 5:**
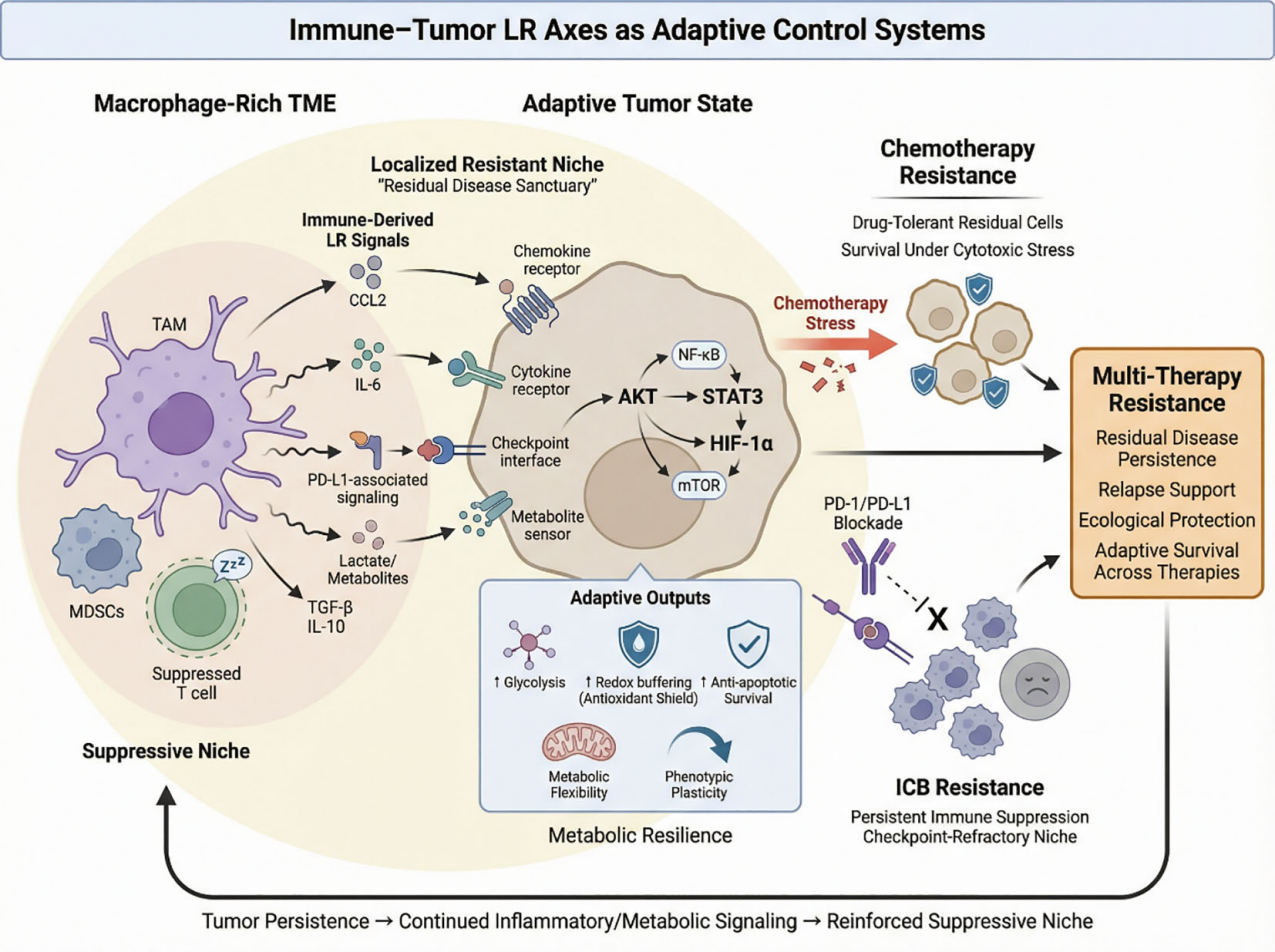
Immune–tumor ligand–receptor circuits establish suppressive niches that promote tumor metabolic resilience, survival signaling, and phenotypic plasticity. These adaptive states support both chemotherapy resistance and immune checkpoint blockade resistance, allowing residual tumor cells to persist and driving multi-therapy resistance.

This view helps reconcile several otherwise fragmented observations: why resistance is often spatially heterogeneous, why it may arise rapidly before stable genetic change is evident, why it can be partially reversible, and why different therapies often fail through overlapping biological programs. If tumor cells are continually instructed by immune-derived ligands and supported by suppressive niches, then therapeutic resistance becomes a property of the tumor ecosystem rather than of the malignant clone alone. Such a perspective also changes how resistance should be targeted. Durable therapeutic benefit may require not only eliminating proliferative tumor cells, but also dismantling the LR circuits that maintain residual disease, metabolic resilience, and immune dysfunction. This is especially important in tumors where therapy itself reshapes the immune microenvironment in ways that favor adaptation. Under those conditions, treatment can inadvertently intensify the very communication networks that support survival. Therefore, immune–tumor LR axes should be regarded as central determinants of therapeutic outcome across treatment modalities. They provide the signaling infrastructure through which inflammation, metabolism, immune suppression, and plasticity are woven into resistant tumor states. The next step, accordingly, is to examine how emerging technologies can map these communication systems *in situ* and help identify the most actionable axes for clinical intervention. **Figure 5** illustrates immune–tumor LR signaling as a unifying framework for therapeutic resistance across treatment modalities. It highlights how suppressive microenvironmental cues generate adaptive tumor states that sustain residual disease, immune evasion, and relapse-supportive niches.

## 7 Technologies for mapping immune–tumor ligand–receptor axes in situ

### 7.1 Single-cell approaches for resolving immune–tumor communication

The expanding recognition that immune–tumor ligand–receptor axes shape metabolic adaptation and therapeutic resistance has created a pressing need for technologies capable of resolving these interactions at high cellular resolution. Bulk transcriptomic and proteomic methods have provided important initial insights into tumor metabolism, immune composition, and inflammatory signaling, but they are fundamentally limited in their ability to define who is signaling to whom. Because ligand–receptor communication is inherently relational, meaningful analysis requires approaches that distinguish individual cell populations, capture their transcriptional states, and infer potential signaling partnerships within complex tissue ecosystems. Single-cell technologies have therefore become central to the study of immune–tumor communication.

Single-cell RNA sequencing (scRNA-seq) has transformed this field by enabling the simultaneous profiling of tumor cells, macrophages, lymphocytes, dendritic cells, fibroblasts, endothelial cells, and other stromal populations within the same lesion (155). This has made it possible to identify putative sender and receiver populations based on ligand and receptor expression patterns, and to reconstruct cellular interaction networks that were previously obscured in bulk measurements. In the context of the present review, scRNA-seq is particularly valuable because it can reveal how metabolically distinct tumor subpopulations are embedded within specific immune signaling environments. For example, one subset of tumor cells may show high glycolytic signatures together with elevated receptor expression for inflammatory ligands, whereas another may display lipid metabolic adaptation associated with checkpoint-linked signaling. Such distinctions are essential for understanding how metabolic plasticity is distributed across the tumor and how it relates to immune niche structure.

A major strength of single-cell approaches is their ability to uncover heterogeneity not only between cell types but also within them. TAMs, for instance, are not a uniform population, nor are tumor cells. Both exist along continua of activation, differentiation, metabolic state, and spatially conditioned function. Single-cell profiling allows these continua to be resolved, making it possible to identify which macrophage subsets express ligand repertoires most associated with tumor survival, which tumor cell subclones preferentially express receptors linked to metabolic adaptation, and whether therapy-resistant cells occupy transcriptional states enriched for communication competence. This level of resolution is particularly important for studying resistance, because resistant phenotypes often emerge from minority subpopulations rather than from the dominant tumor bulk. Another important contribution of single-cell methods is the ability to integrate cell-state information with functional pathway inference. Beyond cataloging ligand and receptor transcripts, computational frameworks can estimate pathway activity, metabolic programs, transcription factor networks, and stress-response modules within each cell. When these layers are analyzed together, they allow a more mechanistic interpretation of immune–tumor LR interactions. A tumor cell population expressing receptors for chemokines or inflammatory cytokines, for example, becomes more biologically meaningful if it also displays increased glycolytic activity, STAT3 signaling, or redox adaptation. In this way, single-cell approaches help connect communication inference to the downstream phenotypes that are central to therapy resistance (156). Despite these advantages, single-cell transcriptomics also has important limitations. The presence of a ligand transcript in one cell type and a receptor transcript in another does not by itself prove functional signaling. Transcriptional abundance may not reflect protein expression, receptor activation, ligand secretion, or effective cell–cell contact. Some ligands are rapidly degraded, some receptors are regulated post-translationally, and many signaling events depend on spatial proximity that conventional scRNA-seq cannot capture. In addition, tissue dissociation may distort fragile cell states or selectively deplete certain populations. These limitations are especially relevant when studying metabolite-associated signaling and checkpoint biology, where receptor activation and membrane localization may matter as much as transcript abundance.

Nevertheless, single-cell approaches provide an indispensable first layer of resolution for mapping immune–tumor communication. They are especially powerful when used to identify candidate LR axes, define cellular states associated with metabolic adaptation, and prioritize interactions for deeper spatial and functional validation. Rather than offering a complete picture on their own, single-cell methods should be seen as foundational tools that generate a high-resolution cellular map upon which more spatially explicit and mechanistically rigorous analyses can be built.

### 7.2 Spatial transcriptomics and imaging-based validation

Although single-cell approaches can identify potential ligand–receptor partnerships, they cannot fully establish whether those interactions occur within the native architecture of the tumor microenvironment. Ligand–receptor signaling is inherently spatial. Cells must be located within appropriate physical range, positioned in compatible tissue niches, and embedded in microenvironments that support effective communication. For this reason, spatial transcriptomics and advanced imaging approaches have become increasingly important for validating and contextualizing immune–tumor LR axes *in situ*.

Spatial transcriptomics adds a crucial dimension to communication analysis by preserving the geographic organization of tissues while capturing gene expression patterns across tumor sections. This allows investigators to determine whether receptor-expressing tumor cells are physically associated with ligand-producing immune populations, whether such interactions are enriched at invasive fronts, hypoxic regions, perivascular zones, or therapy-damaged niches, and whether metabolic programs show spatial concordance with specific immune cell neighborhoods. These capabilities are particularly important for the themes of this review, because metabolic rewiring and therapeutic resistance are rarely distributed uniformly. Instead, they often arise in specialized ecological territories where immune pressure, nutrient stress, and stromal remodeling intersect.

In the study of metabolite-driven LR signaling, spatial methods are especially valuable because they help identify where lactate-rich, immune-suppressive, or checkpoint-enriched niches are located within the tissue. A tumor may contain highly glycolytic subregions adjacent to macrophage-rich zones, areas of T-cell exclusion, or interfaces with dense extracellular matrix. These spatial relationships are not peripheral details; they are central to understanding how communication networks are organized. A putative LR axis identified by transcriptomic inference becomes substantially more compelling when it is localized to a structurally coherent niche in which the necessary sender and receiver populations coexist and the downstream phenotypic consequences are evident.

Multiplex imaging technologies further extend this principle by providing direct visualization of proteins, signaling states, and cellular arrangements within intact tissue. Techniques such as multiplex immunofluorescence, imaging mass cytometry, multiplexed ion beam imaging, and highly multiplexed immunohistochemistry can simultaneously detect immune markers, tumor markers, checkpoint molecules, metabolic enzymes, and signaling mediators at subcellular or single-cell resolution. This makes it possible to confirm that specific ligands and receptors are expressed at the protein level, to examine whether receptor-bearing tumor cells are adjacent to relevant immune subsets, and to determine whether resistant tumor niches exhibit coordinated expression of checkpoint molecules, glycolytic enzymes, macrophage markers, or stress-response signals. Imaging-based validation is particularly important because it bridges the gap between inferred communication and morphologic reality. A tumor cell cluster predicted to respond to macrophage-derived ligands can be directly examined for its proximity to macrophages, its expression of the corresponding receptor, and its associated metabolic phenotype. Similarly, checkpoint-linked metabolic states can be evaluated within the tissue context in which immune exclusion or immune dysfunction occurs. This is essential for avoiding overinterpretation of transcriptomic co-expression alone and for identifying the niche-level architecture that sustains resistant cell populations. Another major advantage of spatial and imaging approaches is their ability to reveal microenvironmental compartmentalization. Tumors frequently contain discrete territories characterized by distinct combinations of inflammation, hypoxia, fibrosis, vascularization, and immune infiltration. These compartments may support different LR networks and different modes of resistance. Some may be dominated by macrophage–tumor communication, others by checkpoint-rich lymphoid exclusion zones, and others by metabolically stressed but sparsely infiltrated tumor clusters. By resolving these compartments directly, spatial technologies help explain why a single tumor can harbor multiple adaptive ecosystems at once.

However, spatial approaches also have limitations. Depending on the platform, they may provide lower gene coverage, lower cellular resolution, or less sensitivity than dissociative single-cell approaches. Imaging-based methods often require prior marker selection, which can bias analysis toward expected pathways rather than discovering new ones. Moreover, spatial proximity still does not automatically prove functional ligand–receptor signaling. Nonetheless, when integrated with transcriptomic and mechanistic data, these methods provide a far stronger basis for interpreting immune–tumor communication than any single approach alone. In the context of therapy resistance, spatial and imaging technologies are especially important because they can identify where residual disease niches are formed, how suppressive immune cells are arranged around resistant tumor cells, and which communication axes are most closely associated with treatment failure *in situ*. These capabilities make them indispensable for moving the field from theoretical interaction maps toward biologically and clinically meaningful models of immune–tumor adaptation.

### 7.3 Multi-omics integration and computational modeling

As the study of immune–tumor ligand–receptor communication becomes more sophisticated, it is increasingly clear that no single data type is sufficient to define the full biology of adaptive resistance. Transcriptomics can suggest potential signaling interactions, spatial approaches can localize them, and imaging can validate their tissue context, but ligand–receptor communication ultimately operates through complex downstream consequences involving proteins, metabolites, signaling cascades, chromatin states, and cellular behaviors. Multi-omics integration and computational modeling have therefore emerged as essential strategies for transforming descriptive datasets into mechanistic insight.

One major advantage of multi-omics integration is that it allows communication axes to be linked to functional tumor states more directly. Transcriptomic evidence of receptor expression becomes more compelling when accompanied by phosphoproteomic evidence of downstream pathway activation, metabolomic evidence of glycolytic or lipid remodeling, proteomic evidence of ligand abundance, or chromatin-based evidence of transcriptional reprogramming. For example, an inferred macrophage-to-tumor cytokine axis gains mechanistic weight if the corresponding tumor cell population shows activated STAT3 or AKT signaling together with increased glycolytic metabolites and stress-adaptive transcriptional programs. Similarly, checkpoint-associated LR axes can be understood more fully when PD-L1 expression is interpreted together with lipid metabolic signatures, membrane-associated signaling proteins, and local immune cell dysfunction.

Metabolomics is particularly important for the subject of this review because metabolic rewiring is a central output of immune–tumor communication. Transcriptomic signatures of glycolysis or lipid metabolism are informative, but they do not always reflect actual metabolic flux or microenvironmental metabolite abundance. Direct measurement of lactate, amino acids, lipids, redox intermediates, and nutrient gradients can reveal whether predicted communication axes are associated with the metabolic states they are hypothesized to induce. When spatial metabolomics or imaging mass spectrometry is added, these analyses become even more powerful, allowing signaling niches to be connected to the biochemical landscapes that sustain them. Proteomic and phosphoproteomic approaches also play a critical role because ligand–receptor biology is often regulated beyond the level of RNA. Surface receptor abundance, phosphorylation status, ligand secretion, proteolytic processing, and receptor internalization can all influence signaling strength without being obvious at the transcript level (157). In therapy-resistant tumors, these post-transcriptional and post-translational layers may be especially important, as drug exposure can rapidly reshape signaling competence independent of gene expression alone. Integrating proteomic information therefore helps distinguish potentially functional LR axes from those that are merely transcriptionally plausible.

Computational modeling is equally important because immune–tumor communication operates as a network rather than a collection of isolated interactions. Modern computational frameworks can infer putative ligand–receptor pairings, score directional communication probabilities, identify dominant sender and receiver populations, and integrate these findings with pathway activity, cell-state transitions, and survival phenotypes. Network-based and causal modeling approaches are especially valuable for identifying higher-order regulatory modules that persist across patients or tumor types. In the setting of therapeutic resistance, such models may reveal that diverse ligands converge on a smaller number of signaling hubs or metabolic endpoints, thereby helping prioritize the most actionable vulnerabilities.

Machine learning and artificial intelligence approaches are beginning to enhance this process further by extracting communication patterns from high-dimensional datasets that would be difficult to interpret manually. These methods may help identify combinations of LR axes associated with relapse, distinguish treatment-responsive from refractory niches, or predict which metabolic programs are most tightly coupled to specific immune states. However, the utility of these models depends heavily on biologic grounding. Predictive strength alone is not sufficient; the resulting communication hypotheses must still be anchored in tissue context, mechanistic plausibility, and experimental validation. At the same time, computational and multi-omics approaches face important challenges. Inference algorithms often depend on curated ligand–receptor databases that may not fully capture context-specific biology, especially for metabolite-associated signaling, noncanonical receptor usage, or post-translationally regulated interactions. Integration across platforms can also be technically difficult because of differences in resolution, scale, and sample processing. Most importantly, computational inference does not replace validation. A communication axis predicted from public or clinical datasets remains a hypothesis until it is supported by spatial evidence, functional perturbation, or independent biological confirmation. Despite these challenges, the integration of single-cell data, spatial analysis, metabolomics, proteomics, imaging, and computational modeling offers the most promising route toward a truly mechanistic map of immune–tumor communication. Rather than treating tumor metabolism, immune signaling, and therapeutic resistance as separate layers, multi-omics approaches make it possible to study them as parts of the same adaptive system. This is particularly valuable for identifying communication axes that are not only biologically important but also clinically actionable.

### 7.4 From interaction maps to actionable biology

The ultimate goal of these technologies is not simply to generate increasingly detailed maps of tumor ecosystems, but to identify which immune–tumor LR axes are functionally decisive for metabolic adaptation and therapeutic resistance. High-resolution maps are essential, but they become clinically meaningful only when they help distinguish passenger interactions from driver circuits. In other words, the challenge is no longer merely to observe communication, but to determine which interactions actually maintain resistant tumor states and therefore represent realistic targets for intervention. Achieving this requires a layered strategy. Single-cell methods can nominate candidate sender–receiver relationships, spatial approaches can localize them, imaging can validate their protein-level organization, and multi-omics integration can link them to downstream metabolic and signaling consequences. Functional perturbation, whether through receptor blockade, ligand neutralization, pathway inhibition, or genetic manipulation, is then needed to test causality. Only by moving through these layers can the field identify which communication axes are robust enough to support biomarker development or rational combination therapy design. This transition from mapping to action is especially important in the context of resistant disease. A therapy-resistant niche may contain dozens of apparent ligand–receptor interactions, but only a subset may actually be required for tumor persistence. Some interactions may reflect inflammatory background noise, whereas others may serve as central organizing systems for metabolic survival, immune suppression, or myeloid recruitment. Technologies that allow these differences to be resolved are therefore critical not only for mechanistic clarity but also for translational prioritization.

Overall, emerging technologies are reshaping how immune–tumor communication is studied. They are moving the field from static catalogs of genes and cell types toward dynamic, spatially grounded, and mechanistically interpretable models of adaptive resistance. In doing so, they provide the foundation for the next major question: how these LR circuits can be translated into biomarkers and therapeutic opportunities capable of improving cancer treatment outcomes.

## 8 Translational implications and therapeutic opportunities

### 8.1 Immune–tumor ligand–receptor axes as biomarkers of adaptive resistance

One of the most important translational implications of immune–tumor ligand–receptor signaling is its potential to serve as a biomarker framework for adaptive resistance. Traditional biomarkers in oncology often focus on tumor-intrinsic features, such as driver mutations, gene amplification, protein overexpression, or broad immune markers like PD-L1 positivity and tumor mutational burden (158). Although these indicators can be clinically useful, they frequently fail to capture the dynamic and microenvironment-dependent nature of treatment response. In contrast, LR axes provide a relational biomarker model, one that reflects not only what the tumor is, but also how it is being instructed by its surrounding immune ecosystem. This distinction is particularly relevant for therapy resistance. A tumor may appear molecularly targetable or immunologically permissive at baseline, yet still fail treatment because immune-derived signals sustain a metabolically adaptive, drug-tolerant state. Biomarkers based on ligand–receptor communication may be better suited to detect such states because they capture active signaling relationships rather than static molecular attributes. For example, the co-occurrence of ligand-producing macrophage subsets with receptor-expressing glycolytic tumor cells may be more informative than either feature alone. Likewise, a tumor in which checkpoint-associated signaling is tightly linked to lipogenic or glycolytic programs may represent a more therapy-refractory state than one with PD-L1 expression in isolation.

An additional advantage of LR-based biomarkers is that they can integrate multiple layers of tumor biology simultaneously. A single communication axis may reflect immune composition, tumor cell plasticity, inflammatory status, and metabolic configuration at once. For instance, a lactate-associated receptor axis may indicate high glycolytic flux, suppressive myeloid recruitment, and impaired antitumor immunity within the same niche. Similarly, a chemokine-driven axis may point to both inflammatory activation and receptor-mediated metabolic adaptation in tumor cells. Such multidimensionality is valuable in resistant disease, where no single molecular variable is usually sufficient to explain outcome. Spatial context further enhances the biomarker potential of LR circuits. The clinical relevance of a signaling axis may depend not only on whether it is present, but on where it is organized within the tissue. A macrophage–tumor survival axis located at the invasive front, around residual viable tumor nests after therapy, or within hypoxic core regions may carry different prognostic or predictive significance than a similar interaction distributed diffusely. As spatial profiling technologies mature, LR biomarkers may therefore evolve from simple molecular readouts into niche-level indicators of resistance risk and therapeutic vulnerability.

Importantly, LR-based biomarkers may also improve patient stratification for combination therapy. If a tumor is found to rely on chemokine-mediated myeloid recruitment, checkpoint-linked metabolic adaptation, or lactate-centered immunosuppressive signaling, these communication states could guide the selection of rational therapeutic partners. In this sense, LR biomarkers are not only descriptive; they are potentially actionable. They may help identify which patients are most likely to benefit from adding a metabolic inhibitor, macrophage-targeting strategy, or chemokine receptor antagonist to standard treatment. However, several challenges remain before such biomarkers can be implemented clinically. First, many LR interactions are context dependent and may vary across tumor type, treatment stage, and tissue region. Second, robust assays are needed to measure ligands, receptors, and associated metabolic states in a standardized and scalable way. Third, biomarker interpretation must move beyond the binary presence or absence of a molecule toward integrated models that incorporate cell-state composition, spatial organization, and pathway activity. Despite these challenges, the concept of LR-based biomarkers represents a promising advance because it aligns more closely with the biology of adaptive resistance than conventional tumor-centric markers alone. Overall, immune–tumor LR axes offer a biomarker paradigm capable of capturing the dynamic interplay between metabolism, immunity, and therapy response. By reflecting the active communication systems that maintain resistant niches, they may enable more precise prediction of treatment outcome and more informed selection of therapeutic strategies.

### 8.2 Therapeutically targeting ligands, receptors, and downstream metabolic dependencies

If immune–tumor LR axes help sustain adaptive resistance, then disrupting these axes represents an attractive therapeutic strategy. The most direct approach is to target the ligands or receptors themselves. In principle, blocking the extracellular communication that supports tumor survival could prevent resistant cells from receiving the inflammatory, metabolic, or immunosuppressive cues required for persistence (159, 160). This strategy is conceptually appealing because it intervenes at the point where the tumor microenvironment exerts instructive control over malignant cell state. Targeting ligands may be particularly useful in tumors dominated by specific myeloid or inflammatory signals. Neutralizing cytokines, interfering with chemokines, or preventing the recruitment of ligand-producing immune subsets could reduce the microenvironmental pressure that drives metabolic adaptation. Likewise, receptor inhibition on tumor cells may interrupt the capacity to sense and respond to these external cues. Such strategies could be especially valuable when the relevant receptor is enriched in resistant tumor subpopulations or is associated with defined metabolic states such as glycolytic adaptation, lipogenic remodeling, or checkpoint-linked survival.

However, direct targeting of ligands and receptors is unlikely to be sufficient in all cases. One reason is redundancy: multiple ligands may converge on similar downstream pathways, and tumor cells may compensate by engaging alternative communication routes. For this reason, downstream signaling nodes and metabolic dependencies represent equally important therapeutic targets. Pathways such as AKT, STAT3, mTOR, HIF-1α, and SREBP act as common executors of many LR-mediated effects, while metabolic programs such as glycolysis, lactate export, fatty acid synthesis, and redox buffering often function as final support systems for resistant phenotypes. Intervening at these downstream levels may collapse the adaptive state even when the upstream LR landscape is heterogeneous. This logic is particularly relevant for lactate-centered signaling. In such settings, potential points of intervention include glycolytic flux, lactate production, lactate transport, receptor sensing, and the suppressive immune consequences of lactate accumulation. Similarly, in checkpoint-linked metabolic circuits, one might combine PD-1/PD-L1 blockade with inhibitors of glycolysis, lipid metabolism, or shared signaling hubs that sustain PD-L1-associated tumor fitness. In chemokine- and cytokine-driven resistance, receptor antagonism could be paired with metabolic or transcriptional inhibition to prevent downstream compensation. These examples illustrate that therapeutic targeting of LR axes should not be viewed as a single-drug strategy, but rather as a framework for designing multi-level intervention.

A further advantage of this framework is that it aligns with the reversible nature of many resistant states. If tumor survival is maintained in part by continuous exposure to microenvironmental signals, then disrupting those signals may destabilize resistance without needing to eliminate every resistant cell directly. In this sense, targeting immune–tumor communication may not only kill tumor cells, but also deprive them of the ecological support systems that preserve drug-tolerant states. This is especially important in minimal residual disease, where the goal may be to collapse supportive niches before they seed relapse. At the same time, therapeutic targeting of LR axes requires caution. Because many of these pathways also operate in normal tissue repair and host defense, systemic blockade may carry toxicity or interfere with beneficial immune functions. Moreover, the same ligand or receptor may have different roles depending on cell type, disease stage, or therapeutic context. These issues underscore the importance of precise patient selection and mechanistically grounded intervention design. The challenge is not simply to inhibit communication, but to disrupt the communication modules most critical for resistant tumor persistence while preserving as much protective immunity as possible.

In summary, therapeutically targeting immune–tumor LR axes can be approached at multiple levels: ligand availability, receptor engagement, downstream signaling convergence, and metabolic dependency. The greatest promise likely lies in combinations that interrupt communication while simultaneously disabling the metabolic adaptations it supports. Such approaches may offer a more effective route to overcoming resistant disease than strategies focused exclusively on tumor-intrinsic vulnerabilities.

### 8.3 Rational combination strategies at the immune–metabolic interface

Because immune–tumor LR signaling and metabolic rewiring are tightly intertwined, rational combination therapy is likely to be more effective than single-axis intervention. Many resistant tumors do not rely on a solitary pathway; instead, they persist through coupled systems in which inflammatory communication, checkpoint signaling, nutrient adaptation, and suppressive niche formation reinforce one another. A therapeutic strategy that targets only one component of this system may produce temporary benefit, but the remaining network often allows resistance to recover. Combination strategies are therefore especially attractive because they aim to dismantle the architecture of adaptation rather than merely inhibit one node within it.

One broad strategy is to combine immune checkpoint blockade with metabolic intervention. If PD-L1 expression is supported by glycolytic or lipogenic programs, or if PD-L1 itself contributes to metabolic adaptation, then targeting tumor metabolism may enhance the efficacy of checkpoint blockade by weakening both tumor survival and immune resistance simultaneously. Such combinations may be especially relevant in tumors with glycolysis-high, lactate-rich, or lipid-remodeled phenotypes, where checkpoint inhibition alone may fail because the tumor remains metabolically protected even after immune reactivation. A second strategy is to combine myeloid-targeting approaches with metabolic or checkpoint-directed therapy. Macrophages and other myeloid populations are central sources of chemokines, cytokines, and suppressive signals that shape tumor metabolic survival. Reducing their recruitment, reprogramming their phenotype, or blocking the ligands they produce could diminish the support systems that allow tumor cells to endure therapy. Pairing this with chemotherapy, targeted therapy, or checkpoint blockade may be particularly effective in tumors where macrophage-rich niches correlate with residual disease and relapse.

A third strategy is to combine ligand/receptor blockade with downstream pathway inhibition. Because many LR axes converge on signaling hubs such as AKT, STAT3, HIF-1α, or mTOR, blocking the receptor alone may not fully suppress adaptation if downstream pathways remain active through compensatory signals. Conversely, targeting only a central pathway may be less effective if upstream communication continues to reinforce the resistant niche. Combinations that intervene at both levels may therefore produce more durable disruption of tumor adaptation. Such a design is conceptually attractive because it addresses both the source of instruction and the machinery that executes it. A fourth strategy involves targeting residual disease niches directly. Following chemotherapy, radiotherapy, targeted therapy, or immunotherapy, the surviving tumor fraction is often maintained by specific microenvironmental support systems (161, 162). These niches may be characterized by lactate accumulation, macrophage enrichment, checkpoint-associated signaling, or inflammatory chemokine gradients. Rather than waiting for overt relapse, combination therapy could be designed to specifically collapse these residual ecosystems. This approach may be particularly important in adjuvant or consolidation settings, where the disease burden is low but the biologic risk of relapse remains high. The success of such strategies will depend heavily on biomarker-guided selection. Not all tumors rely on the same communication circuits, and not all patients will benefit from the same combinations. Some tumors may be dominated by lactate-centered immunosuppression, others by macrophage-derived cytokine support, and others by checkpoint-linked metabolic adaptation. The challenge is therefore to align therapy with the dominant LR architecture of each tumor rather than applying uniform combinations indiscriminately. This again underscores the value of single-cell, spatial, and multi-omics approaches for defining clinically meaningful communication states.

Taken together, rational combination strategies at the immune–metabolic interface represent one of the most promising translational directions emerging from this field. By simultaneously disrupting immune-derived signals and the metabolic programs they sustain, such approaches may weaken the adaptive foundations of therapeutic resistance more effectively than conventional monotherapies.

### 8.4 Current challenges and future directions in clinical translation

Despite the conceptual appeal of targeting immune–tumor LR axes, several major challenges must be addressed before this framework can be translated effectively into clinical practice. One challenge is context dependency. The same ligand–receptor pair may have distinct effects depending on tumor type, cellular composition, disease stage, prior treatment exposure, and spatial niche. A signaling axis that promotes resistance in one setting may be less relevant, or even functionally different, in another. This means that therapeutic strategies based on LR biology will require careful contextualization rather than broad generalization. A second challenge is intratumoral heterogeneity. Resistant tumors often contain multiple coexisting communication ecosystems, not all of which are equally important for disease persistence. Some niches may be highly glycolytic and macrophage enriched, whereas others may be more checkpoint dominant or structurally immune excluded. This diversity complicates both biomarker development and target selection. Clinical translation will therefore depend on identifying which LR circuits are true drivers of relapse and which are secondary features of broader tissue remodeling. A third challenge is dynamic evolution during therapy. Immune–tumor communication is not static. Treatments themselves alter immune composition, stromal structure, metabolite gradients, and receptor expression. As a result, a communication axis that is important before treatment may not be the same one that governs residual disease or acquired resistance later. Longitudinal sampling and adaptive biomarker strategies will be essential for capturing this evolving biology. Without them, therapies may be directed against communication states that are no longer dominant by the time resistance becomes clinically evident.

A fourth challenge concerns functional validation and druggability. Many inferred LR axes are biologically plausible, but only a subset will prove functionally necessary for maintaining resistant tumor states. Even among those that are important, not all will be easily druggable. Some may involve broadly expressed receptors, redundant ligands, or context-specific signaling mechanisms that are difficult to inhibit selectively. Future work will therefore need to prioritize not only biologic relevance but also therapeutic tractability. Finally, there is the challenge of integrating complex data into clinically actionable decisions. Single-cell sequencing, spatial transcriptomics, multiplex imaging, metabolomics, and computational modeling generate rich but complex datasets that are not yet easily incorporated into routine oncology workflows. For LR-based translational strategies to succeed, these data will need to be distilled into robust, interpretable biomarkers and treatment rules that can be applied across institutions and patient populations. Despite these obstacles, the future of this field is highly promising. The convergence of tumor immunology, cancer metabolism, and spatial systems biology is creating a far more integrated understanding of treatment resistance than was previously possible. As technologies mature and mechanistic insights deepen, immune–tumor LR axes are likely to become increasingly important not only as explanatory models of resistance, but also as practical guides for biomarker development and therapeutic design. Overall, the translational potential of this field lies in its ability to redefine resistant cancer as a problem of maladaptive communication rather than tumor autonomy alone. By identifying and disrupting the signaling systems that connect immune pressure to metabolic survival, future therapies may be better positioned to eliminate residual disease and achieve more durable treatment responses. As shown in **Table 3**, immune–tumor LR axes can be therapeutically targeted at multiple levels, including ligand availability, receptor engagement, downstream signaling convergence, and metabolic dependency. This framework supports biomarker-guided combination strategies aimed at dismantling resistant tumor ecosystems rather than targeting tumor cells alone.

**Table 3:** Translational opportunities for targeting immune–tumor ligand–receptor axes in resistant cancers.

Translational level	Example target class	Rationale	Expected therapeutic effect	Main challenge
Ligand targeting	CCL2, IL-6, lactate-associated signaling	Reduce immune-derived or metabolite-driven instruction of resistant tumor states	Weaken external survival cues and suppressive niche formation	Ligand redundancy and context dependence
Receptor targeting	CCR2, IL-6R, GPR81/HCAR1, checkpoint-related receptors/interfaces	Block tumor sensing of inflammatory or metabolic signals	Impair adaptive signaling and niche responsiveness	Receptor expression heterogeneity
Downstream pathway targeting	AKT, STAT3, HIF-1α, mTOR, SREBP-associated programs	Interrupt convergent signaling hubs activated by multiple LR axes	Collapse shared metabolic survival programs	Pathway compensation and systemic toxicity
Metabolic dependency targeting	HK2, PKM2, lactate transporters, lipid metabolism regulators	Exploit the metabolic liabilities created by LR signaling	Sensitize tumors to chemotherapy or immunotherapy	Metabolic plasticity and normal tissue effects
Niche-directed combination therapy	Checkpoint blockade + metabolic inhibitor; myeloid targeting + chemotherapy; chemokine blockade + ICB	Resistant disease is maintained by communication circuits rather than isolated pathways	More durable disruption of residual disease ecosystems	Need for biomarker-guided patient selection
Biomarker development	Spatial LR signatures, macrophage–tumor interaction patterns, checkpoint-metabolic coupling states	Identify tumors reliant on specific communication modules	Better stratification for rational combination therapy	Standardization and validation across cohorts

## 9 Conclusions and future perspectives

Therapeutic resistance is increasingly recognized as an ecosystem-level process rather than a purely tumor-cell-intrinsic event. Immune–tumor ligand–receptor (LR) axes provide a useful framework for understanding how extracellular immune cues, metabolite-associated signals, and receptor-linked intracellular pathways may contribute to metabolic reprogramming, immune evasion, phenotypic plasticity, and treatment tolerance. By connecting inflammatory cytokines, chemokines, lactate-centered signaling, checkpoint-associated pathways, and downstream metabolic programs, LR-based communication may help explain why resistant tumor states are spatially heterogeneous, dynamically remodeled by therapy, and sometimes partially reversible.

Future studies should move beyond descriptive interaction maps and prioritize spatially resolved, functionally validated, and clinically actionable models of immune–tumor communication. First, spatial validation will be essential. Candidate LR axes inferred from single-cell or computational analyses should be confirmed within intact tissue using spatial transcriptomics, multiplex immunofluorescence, imaging mass cytometry, spatial metabolomics, or related approaches. Such validation can determine whether ligand-producing immune cells, receptor-expressing tumor cells, metabolic programs, and resistant niches are truly co-localized *in situ*. Second, dynamic sampling before treatment, during therapy, and at relapse should be incorporated whenever possible, because the dominant LR–metabolic programs that sustain residual disease may differ from those present at baseline. Longitudinal profiling may clarify how therapy reshapes immune composition, ligand availability, receptor expression, metabolite gradients, and downstream signaling states.

Third, multi-omics integration will be required to connect predicted communication axes with functional biology. Transcriptomic evidence of ligand–receptor expression should be interpreted together with proteomics, phosphoproteomics, metabolomics, spatial information, and metabolic flux data. This is particularly important because mRNA expression alone cannot establish ligand secretion, receptor activation, protein-level signaling, or metabolic rewiring. Fourth, functional perturbation experiments are needed to distinguish driver LR circuits from passenger associations. Co-culture systems, organoid–immune cell models, ligand neutralization, receptor blockade, genetic manipulation, isotope tracing, and *in vivo* models should be used to test whether specific LR axes are required for metabolic adaptation, immune suppression, and therapeutic resistance.

Finally, these mechanistic insights should guide rational combination therapy design. Rather than targeting isolated pathways, future strategies should aim to disrupt both immune–tumor communication and the metabolic dependencies maintained by that communication. Examples may include combining checkpoint blockade with lactate or lipid metabolic interventions, pairing myeloid-targeted therapy with cytokine or chemokine receptor inhibition, or integrating LR-axis blockade with chemotherapy, targeted therapy, or radiotherapy in tumor-type-specific settings. Importantly, such approaches should be guided by the dominant LR architecture of each tumor rather than applied as uniform pan-cancer combinations. Overall, a future framework that combines spatial validation, longitudinal sampling, multi-omics integration, functional perturbation, and rational therapeutic design may help translate immune–tumor LR biology into more precise strategies for overcoming adaptive resistance.
